# Detecting FIMI: A methodological framework for testing OSINT tools in TTPs detection

**DOI:** 10.12688/openreseurope.21702.1

**Published:** 2026-05-09

**Authors:** Konstantinos Margaros, Theodoros Paparrigopoulos, Georgios Giataganas, Orfeas Filippopoulos, Nikolaos Georgiou, Oleksandr Manzhai, Panagiota Benekou

**Affiliations:** 1 Center for Security Studies (KEMEA), Athens, 10177, Greece; 2 Hellenic Police, Athens, Greece; 3 Kharkiv National University of Internal Affairs, Kharkiv, Ukraine

**Keywords:** FIMI, disinformation, OSINT, TTP detection, DISARM, AMITT, threat intelligence, tool evaluation

## Abstract

**Background:**

Foreign Information Manipulation and Interference (FIMI) represents a growing hybrid threat targeting democratic processes, social cohesion, and diaspora communities. Detecting such operations requires identifying underlying tactics, techniques, and procedures (TTPs) described in frameworks such as DISARM, AMITT, and MITRE ATT&CK. Although numerous open-source intelligence (OSINT) tools exist, their evaluation remains fragmented and lacks methodological standardisation. This article addresses this gap by presenting a structured framework for testing OSINT tools for TTP detection, developed within the Horizon Europe project RESONANT.

**Methods:**

The methodology integrates three components: (1) a systematic multi-source collection of OSINT tools informed by academic, operational, and grey literature; (2) the creation of synthetic datasets representing text-based, multimedia, network, and infrastructure-level manipulation techniques derived from established FIMI taxonomies; and (3) controlled testing conducted by law enforcement partners from Greece and Ukraine. Tools were classified into eight categories and evaluated using predefined indicators covering accuracy, usability, interoperability, coverage of adversarial behaviours, and sustainability.

**Results:**

The framework enabled reproducible, ethically compliant testing across tool categories. Findings showed strong performance in content-verification tools (fact-checking and multimedia forensics) and infrastructure-level verification (IP, email, and phone lookups). Social media analytics tools were effective in identifying coordination patterns but varied significantly in usability and exportability. Cross-cutting shortcomings included limited multilingual processing, poor cross-platform interoperability, and difficulties integrating outputs into structured threat-intelligence formats. Tool performance was also affected by API volatility, software updates, and platform policy changes.

**Conclusions:**

The study provides a reproducible and operationally grounded methodology for evaluating OSINT tools in the context of FIMI TTP detection. By combining synthetic datasets, structured testing, and practitioner validation, it strengthens methodological transparency and supports the optimisation of tool suites for researchers and law enforcement agencies.

## 1. Introduction

FIMI has emerged as a strategic hybrid threat undermining democratic resilience, electoral integrity, and societal cohesion. Recent reports by the European External Action Service ^1, 2^ identify FIMI as a growing transnational phenomenon that exploits information environments to manipulate perceptions and disrupt decision-making processes. Similar concerns are echoed in the Canadian Special Report on FIMI, which highlights the vulnerabilities of democratic institutions and the role of hostile actors in exploiting information ecosystems. ^3^ Beyond the Euro-Atlantic sphere, the spread of manipulative content in fragile contexts, including Africa and the Middle East, further illustrates the global salience of these activities.

To study and counter FIMI effectively, the focus must extend beyond narratives to the TTPs used by threat actors. TTPs provide structured analytical entry points for identifying patterns of disinformation and information suppression, enabling the detection of recurrent methods across incidents. Frameworks such as the DISARM Navigator and AMITT (Adversarial Misinformation and Influence Tactics and Techniques) have been developed to classify and systematize such activities, mirroring the structured approach of MITRE ATT&CK in cybersecurity. ^4^ These taxonomies enable practitioners to map incidents onto standardised phases of disinformation operations, such as content creation, amplification, suppression, and mobilization, facilitating cross-case comparison and countermeasure design.

Despite these advances, the detection ecosystem remains fragmented. Numerous OSINT and social media analytics tools exist, ranging from event detection systems ^5^ and topic discovery through social network analysis ^6^ to bot detection ^7^ and multimedia verification. Yet, these tools are rarely interoperable and often lack systematic integration with structured TTP frameworks. In addition, significant challenges persist in terms of multilingual coverage, real-time detection, and the incorporation of detection outputs into attribution processes. The problem is compounded by the ethical and legal constraints of data collection, including privacy obligations and the need for data protection impact assessments (DPIAs) in high-risk processing. ^8^

Attribution further illustrates the centrality of detection capabilities. As Sabir ^9^ argues, credible deterrence in cyberspace hinges on reliable attribution; yet anonymity, proxies, and false flag operations frequently undermine accountability. Tsagourias and Farrell ^10^ distinguish between technical, political, and legal attribution, stressing that technical findings, based on indicators such as infrastructure, malware, and tradecraft, rarely translate directly into state responsibility under international law. Tools for TTP detection therefore play a pivotal but partial role: they enable technical attribution by producing observables and indicators that can feed into higher levels of attribution, but they cannot independently resolve responsibility gaps.

Against this backdrop, the Horizon Europe project RESONANT seeks to strengthen the detection and analysis of FIMI operations through an integrated methodological framework. RESONANT provides the first systematic state-of-the-art review of tools for TTP detection, classifying them by function and mapping their coverage against established frameworks (DISARM, AMITT, MITRE ATT&CK). The purpose of this article is to present the results of this review in a scientific format, situating them within the broader academic and policy literature. By consolidating a fragmented tool landscape and linking it with structured taxonomies, the study contributes to building a methodological foundation for testing and validation activities in RESONANT and beyond.

## 2. Background and conceptual framework

### 2.1 TTP frameworks

The concept of **TTPs** originates from cybersecurity and military doctrine, where it is used to capture the observable actions of adversaries across different stages of an operation. In the context of FIMI, TTPs provide a structured way of describing and classifying how disinformation and information suppression campaigns are conducted, from strategic planning to tactical dissemination. By focusing on the *“how”* rather than the *“what”* of disinformation, TTP frameworks enable analysts to identify recurring patterns, compare incidents, and design countermeasures. ^4^

Several frameworks have been adapted to the information environment. The DISARM Navigator is one of the most widely used taxonomies for describing disinformation incidents. It structures cases across dimensions such as actor types, objectives, channels, languages, audiences, and tactics, techniques, and procedures. Its modular design supports both researchers and practitioners by enabling incident coding, cross-case comparison, and the design of countermeasures. DISARM has been adopted by the EEAS and StratCom units for structured incident reporting. ^1^

Complementing this, the Adversarial Misinformation and Influence Tactics and Techniques (AMITT) framework extends the MITRE ATT&CK approach to the domain of information manipulation. It provides a comprehensive catalogue of adversarial behaviors in the information space, organised into operational phases such as planning, seeding, amplification, and suppression. Each phase is linked with specific techniques (e.g., *create fake experts, distort facts, coordinate amplification*) and mapped to potential countermeasures. ^11, 12^ This makes AMITT particularly suitable for operationalising TTP detection, as it provides direct linkages between observed behaviors and recommended responses.

The MITRE ATT&CK framework itself, originally designed for adversary emulation in cybersecurity, remains influential in shaping the terminology and structure of TTP-based approaches. Its emphasis on standardized categorisation and machine-readable formats (e.g., STIX 2.0) has informed the development of both AMITT and DISARM. In practice, many threat intelligence platforms (e.g., OpenCTI, MISP) already integrate ATT&CK, making interoperability with AMITT and DISARM both technically feasible and desirable. ^13^

Together, these frameworks provide the theoretical and methodological backbone for analysing FIMI operations. They enable a shift from narrative-centric approaches to indicator- and procedure-based detection, which is critical for technical attribution. However, as highlighted by Tsagourias and Farrell, ^10^ while such frameworks strengthen the technical dimension of attribution, their outputs must be complemented by political and legal processes to translate into accountability. The mapping of tools in RESONANT is therefore explicitly aligned with DISARM, AMITT and ATT&CK to ensure analytical consistency, operational relevance and future interoperability.

### 2.2 Social media manipulation

The diffusion of manipulative content across social media platforms is a defining feature of FIMI. Social media systems amplify adversarial tactics by lowering the barriers of entry for influence operations and enabling rapid, large-scale dissemination of content. ^14^ The unique affordances of social networks, interactivity, algorithmic curation, and virality, create environments where malicious actors can strategically exploit platform dynamics to distort information ecosystems. ^15^

One of the most studied vectors of manipulation involves the use of automated accounts or social bots. These entities are designed to mimic human behavior, artificially inflating the visibility of certain narratives and creating false perceptions of consensus. Ferrara et al. ^7^ demonstrated that social bots can coordinate content amplification, hijack trending topics, and foster the illusion of grassroots mobilization, a phenomenon often described as *astroturfing.* Their findings highlight how bot-driven activity alters both the speed and scale of disinformation diffusion.

Beyond automation, manipulation also relies on the dynamics of rumor propagation. Qin et al. ^16^ proposed a multi-layered communication model illustrating how rumors spread and stop across social media networks. The model shows that actors strategically seed narratives within clusters of susceptible users, which can escalate to broader networks through re-sharing, while counter-rumors or debunking interventions act as dampening mechanisms. This illustrates the interplay between adversarial tactics and countermeasures in shaping the trajectory of contested information.

Another vector is the use of soft propaganda techniques. Mattingly and Yao ^17^ argue that persuasion in authoritarian and semi-authoritarian contexts increasingly relies on subtle framing, selective omission, and positive reinforcement rather than overt censorship. By embedding messages into entertainment formats or everyday online discourse, propagandists achieve persuasion with reduced resistance. This aligns with earlier observations that effective manipulation often combines truthful elements with misleading or distorted framing, making detection and classification more complex.

Empirical studies have further documented manipulation through case-based evidence. The *Media Manipulation Casebook* catalogues incidents where actors have exploited platform algorithms, influencer networks, and cross-platform coordination to spread disinformation. Examples range from conspiracy amplification during public health crises to adversarial exploitation of geopolitical conflicts. Such cases demonstrate that manipulation is rarely confined to a single tactic or platform; rather, it is characterized by hybridisation, opportunism, and adaptability.

Taken together, these insights underscore that social media manipulation operates through a combination of technical automation, communicative dynamics, and narrative persuasion. For detection purposes, this complexity necessitates the integration of multiple methodological approaches, including network analysis, rumor detection algorithms, bot identification, and content classification. Within RESONANT, these insights are used to inform the mapping of tools capable of addressing manipulation at different layers, technical, social, and cognitive.

### 2.3 OSINT evolution: Trends and security implications

OSINT has become a cornerstone of contemporary approaches to detecting FIMI. Initially conceptualised as a complementary discipline to traditional intelligence, OSINT has evolved into a primary source of indicators and observables for mapping adversarial TTPs. Its evolution reflects both technological advances and the operational needs of law enforcement, civil society, and security institutions.

Several studies highlight the trajectory of OSINT from manual collection and verification to increasingly automated, AI-enabled workflows. Dokman and Ivanjko ^18^ identify key challenges and trends, including data overload, source validation, and the growing reliance on social media platforms as primary arenas for intelligence collection. They emphasise that while OSINT offers opportunities for transparency and accountability, its reliability depends on methodological rigour and the ability to distinguish authentic from manipulated signals.

The role of OSINT in security contexts has expanded significantly with the proliferation of digital traceability. Hwang et al. ^19^ outline how the use of OSINT for cybersecurity and influence operation detection has become institutionalised in both public and private sectors. They describe OSINT as simultaneously a security enabler and a risk factor, since adversaries can also exploit open data to profile targets, craft tailored disinformation, or conduct reconnaissance for hybrid operations. This dual-use character requires refined operational standards and ethical safeguards.

At the EU level, institutional frameworks increasingly promote OSINT as a structured component of FIMI monitoring. The EEAS OSINT Guidelines for FIMI Detection ^2^ set methodological standards for incident documentation, indicator coding, and interoperability with frameworks such as DISARM and AMITT. These guidelines stress the importance of cross-platform monitoring, structured reporting, and alignment with legal and ethical standards, including the General Data Protection Regulation (GDPR).

The implications of these developments are twofold. First, OSINT contributes directly to technical attribution, supporting the provision of evidence such as metadata, network traces, and content analysis that can support the identification of adversarial actors and methods. Second, OSINT serves a democratic accountability function, enabling independent verification of manipulative campaigns by journalists, researchers, and civil society organisations. However, the increasing reliance on OSINT raises critical issues around data protection, source protection, and misinformation contamination. As Demetzou ^8^ argues, accountability in high-risk data processing requires systematic Data Protection Impact Assessments (DPIAs) to ensure compliance and mitigate risks of overreach.

In the context of RESONANT, OSINT is recognised as a central detection layer for mapping TTPs, but its effectiveness depends on the interoperability of tools, the robustness of methodologies, and adherence to ethical standards. This dual imperative, operational efficacy and accountability, frames OSINT as both a technical and normative cornerstone of contemporary FIMI detection.

## 3. Methodology

The methodology applied in RESONANT was designed to provide a structured and reproducible process for testing open-source tools relevant to the detection of TTPs associated with FIMI. The process was inspired by the Waterfall model used in software engineering, ensuring a step-by-step workflow where each stage builds upon the outputs of the previous one. This approach enabled systematic evaluation while maintaining transparency and accountability.

### 3.1 Research requirements and tool categorisation

The first step in the RESONANT methodology was the identification of research requirements, which determined both the scope of the review and the categories of tools to be examined. Rather than approaching tool selection ad hoc, the process was anchored in established taxonomies such as DISARM, AMITT, and MITRE ATT&CK, which systematise adversarial behaviours in information operations. Aligning tool functions with these frameworks ensured that the review addressed the full spectrum of TTPs relevant to FIMI detection, from content creation and manipulation to amplification, suppression, and attribution. ^18, 20^

To complement this conceptual anchoring, consultations with practitioner partners, including the Hellenic Police (HP) and the Kharkiv National University of Internal Affairs/Ministry of Internal Affairs of Ukraine (KhNUIA/MoIA), were conducted. Their input helped refine the selection by highlighting which tools were most relevant to investigative workflows and law enforcement priorities. This combination of framework-driven classification and practitioner validation distinguished the methodology from purely academic reviews, embedding operational relevance into the research design. ^21, 22^

As a result, tools were grouped into eight functional categories: (a) fact-checking platforms, (b) website credibility assessment tools, (c) bot detection tools, (d) image and video analysis tools, (e) geo-IP verification tools, (f ) phone-related tools, (g) social media analysis tools, and (h) email verification tools. Together, these categories reflect three complementary layers of detection: content-level verification (fact-checking, multimedia forensics), platform-level monitoring (social media and bot analysis), and infrastructure-level attribution (IP, phone, and email verification). This layered approach provided the foundation for structured testing and facilitated the identification of complementarities and gaps across the detection ecosystem. ^23– 25^

### 3.2 Dataset preparation and testing environment

A central methodological challenge in evaluating detection tools was the need to replicate realistic FIMI scenarios without relying on live operational data, which would have raised ethical, legal, and security concerns. To address this, synthetic datasets were developed to simulate common manipulation techniques across text, multimedia, social media, and infrastructure layers. These datasets included fabricated narratives derived from known disinformation cases, manipulated and AI-generated images, simulated bot activity, and spoofed infrastructure elements such as disposable email domains, IP addresses, and phone numbers. This practice follows established OSINT methodologies, where controlled simulation is used to mitigate risks linked to privacy and disinformation while preserving analytical validity. ^22, 23^

The use of synthetic data served two purposes. First, it ensured compliance with data protection and ethical obligations, eliminating risks associated with processing personal or sensitive information. Second, it provided a reproducible baseline for testing, as each tool within a given category was exposed to identical inputs, allowing for meaningful comparison of performance. Such reproducibility is a recognised methodological requirement in OSINT and intelligence research, ensuring comparability across tools and datasets. ^18, 20^

To support the evaluation, a dedicated testing environment was established with controlled access for project partners. This environment incorporated detailed logging functions that captured tool outputs, system responses, and operator observations. By reproducing investigative workflows under controlled conditions, the environment enabled stress-testing of tools not only for technical accuracy but also for usability and reliability. Thus, the dataset design and testing environment together ensured that the evaluation was transparent, comparable, and transferable, providing a robust basis for subsequent analysis. The design of this environment drew inspiration from sequential, waterfall-derived approaches that emphasise stepwise validation and traceability, an approach recommended where reproducibility and transparency are paramount. ^26– 30^

### 3.3 Execution of testing

The third stage of the methodology involved the structured execution of the tool testing process. This stage was carried out collaboratively by cybercrime specialists from HP and the KhNUIA/MoIA, ensuring that the exercise combined methodological rigour with operational expertise. Each tool was tested under controlled conditions, using the synthetic datasets prepared in the previous stage. The design of the scenarios was guided by adversarial behaviours drawn from the AMITT and DISARM frameworks, thereby ensuring that testing was directly aligned with recognised phases and techniques of information manipulation. ^18, 20^ The execution process resembled a step-by-step laboratory procedure. Tools within a given category were applied to the same dataset, allowing for direct comparison of outputs. For example, fact-checking platforms were evaluated by inputting identical claims derived from synthetic narratives, while image analysis tools were tested against the same set of manipulated and AI-generated visuals. In the case of social media analysis tools, the same simulated account activity was used to assess whether tools could detect signs of automation, coordination, or amplification. Such structured comparisons are in line with OSINT methodology literature, which stresses the need for reproducibility and transparency in testing environments. ^21, 23^ By maintaining a consistent dataset across tools, the testing process generated results that were both reproducible and comparable. Law enforcement partners played a crucial role in the execution. Their task was not limited to running tools mechanically but also to interpreting outputs in light of investigative practice. For instance, when image alteration detection tools returned visual heatmaps or statistical anomalies, the evaluators assessed not only whether manipulation was correctly identified but also whether the presentation of results was clear enough to be actionable in real investigations. This emphasis on operational usability reflects broader findings in OSINT research, which highlight that actionable intelligence depends as much on clarity and accessibility as on technical accuracy. ^22, 24^ This operational perspective enriched the testing by highlighting usability factors, such as the clarity of interfaces, the transparency of scoring systems, and the extent to which outputs could be easily integrated into broader workflows. Throughout the testing phase, detailed logs were kept of tool performance. These logs included successful detections, errors, false positives, and instances where tools failed to return any result. Recording such outcomes was essential for evaluating reliability and identifying recurring weaknesses within categories. It also provided insight into the dependency of certain tools on external factors such as internet connectivity, API availability, or server stability. The need to document such limitations has also been underlined in studies that analyse systemic OSINT challenges, including the volatility of access to APIs and the instability of online infrastructures. ^18^ The comprehensive documentation of results ensured that the subsequent analysis would not be limited to headline performance but would also reflect the practical challenges encountered during real use. Thus, the execution stage generated the empirical data necessary for performance analysis and replicated, in a controlled setting, the type of workflows that law enforcement and practitioners might follow in operational contexts.

### 3.4 Performance analysis

The final stage of the methodology consisted of analysing the outputs generated during testing, with the aim of assessing the strengths and weaknesses of each tool category. This stage measured whether tools produced correct or incorrect results, considering also factors such as usability, interoperability, and sustainability. In this sense, the analysis sought to capture the multidimensional nature of tool performance, reflecting both technical accuracy and the practical realities of investigative work. ^18, 21^

Performance was evaluated against a set of predefined criteria, treated as key performance indicators (KPIs). Accuracy was examined by comparing the tool’s outputs with the expected results embedded in the synthetic datasets. For instance, in the case of manipulated images, accuracy referred to whether a tool successfully detected the alteration or misclassified the visual as authentic. Usability was assessed through the experience of the law enforcement testers, who recorded their observations on interface clarity, ease of operation, and the learning curve required for effective use. Interoperability referred to the extent to which outputs could be exported or integrated into broader analytical environments, such as open-source threat intelligence platforms. Coverage measured the alignment of each tool with specific adversarial behaviours drawn from frameworks like AMITT and DISARM, thereby assessing not just functionality but relevance to structured TTP detection. Finally, sustainability reflected whether tools were regularly updated, maintained active support communities, or showed signs of obsolescence, such as outdated interfaces or broken data pipelines. ^22, 24, 25^

The evaluation process was iterative, involving both quantitative and qualitative elements. Quantitative measures were derived from the detection results themselves, such as success rates in identifying falsified content or accuracy in classifying suspicious domains. Qualitative insights were drawn from the logs maintained by the evaluators, who noted recurring errors, inconsistencies or usability issues. This dual approach is consistent with recent OSINT methodology studies, which emphasise that actionable intelligence emerges from combining measurable accuracy with user-centered evaluation. ^20, 23^ This combination provided a balanced picture of performance, acknowledging that even tools with high detection accuracy may still fall short if their outputs are opaque or difficult to operationalise.

A further dimension of the analysis involved cross-comparison across categories. Since each category could be targeting different phases of FIMI operations, the analysis highlighted complementarities as well as gaps. For example, fact-checking tools proved strong at identifying recycled narratives but offered little support for technical attribution, while infrastructure verification tools contributed to attribution but could not address content falsification. Similar observations have been made in the literature, where the integration of heterogeneous OSINT tools is seen as key to offsetting categorical weaknesses. ^18, 22^ The recognition of such complementarities was central to the construction of an optimised suite of tools, where individual strengths could be combined to offset categorical weaknesses.

By structuring the analysis in this way, the methodology ensured that findings were both descriptive and actionable. The performance assessment thus served as the bridge between the tool descriptions presented in Section 4 and the evaluative insights consolidated in Section 5, providing a systematic basis for determining which tools could be prioritised in the RESONANT suite and how the methodology could be applied to future detection instruments.

### 3.5 Ethical and legal safeguards

All testing activities were conducted in line with the GDPR and under the project’s DPIA. By using synthetic datasets and open-source tools, the methodology avoided high-risk data processing. Particular attention was paid to the principle of proportionality, ensuring that evaluation focused strictly on tool functionality without unnecessary data collection. The approach was also guided by ethics-by-design principles, ensuring that AI- and NLP-based detection methods were assessed with attention to transparency, explainability and potential bias.

## 4. Results – Tool descriptions and mapping

The state-of-the-art review conducted in RESONANT identified a diverse set of tools relevant to the detection of TTPs linked to FIMI. To ensure analytical clarity, tools were grouped into functional categories, each corresponding to specific detection needs within frameworks such as AMITT and DISARM. The categorisation does not imply exclusivity, as many tools provide overlapping functionalities, but it highlights their principal contributions to different phases of adversarial operations. These categories include fact-checking tools, website credibility assessment tools, bot detection tools, image and video analysis tools, geo-IP verification tools, phone-related tools, social media analysis tools, and email verification tools. The following subsections provide descriptions of each category, including the core functionalities of representative tools and their relevance to TTP detection.

### 4.1 Fact-checking tools

Fact-checking tools represent the first line of defence in countering disinformation and identifying the recycling of debunked claims, a common tactic in FIMI campaigns. These tools allow investigators to cross-reference contested information against repositories of verified claims, thereby distinguishing between authentic and manipulative content. Their role extends beyond validating individual statements; they also enable the identification of recurrent adversarial procedures, such as the strategic reintroduction of false narratives into the information environment. This aligns with AMITT phases related to content creation and seeding, where adversaries exploit old or partially true claims to reinforce manipulative campaigns.

The utility of fact-checking tools lies in their accessibility and their ability to provide a rapid triage mechanism, aggregating claim reviews from multiple certified fact-checking organisations, enabling cross-validation of suspicious narratives, maintaining archives of debunked stories that often resurface during electoral cycles or crises or extending fact-checking into multimedia domains by combining keyword search with basic forensic analysis.

From a TTP detection perspective, fact-checking tools support the early identification of adversarial content falsification and repurposing techniques. They are particularly relevant for distinguishing between deliberate manipulative activity and genuine errors in reporting, an important distinction for law enforcement and policymakers. Nevertheless, RESONANT highlighted limitations: reliance on existing fact-checks can lead to gaps when novel narratives emerge, and the uneven geographical coverage of fact-checking initiatives leaves blind spots in certain languages and regions. Despite these constraints, fact-checking tools remain indispensable as an entry point into the layered detection process, feeding subsequent stages of investigation that rely on multimedia forensics, social media analytics, and threat intelligence platforms.

### 4.2 Website credibility assessment tools

Website credibility assessment tools extend verification practices from individual claims to the evaluation of entire online domains. Rather than focusing on the truthfulness of a single piece of content, these tools analyse and rate the broader information environment in which narratives circulate. RESONANT highlighted several representative tools, which provide structured evaluations of websites on parameters such as transparency of ownership, sourcing practices, adherence to journalistic standards and history of publishing disinformation.

The value of these tools for FIMI detection lies in their ability to flag low-credibility outlets that frequently serve as vectors for adversarial narratives. In many campaigns, content amplification relies on networks of fringe or pseudo-news websites that are designed to mimic legitimate media outlets while promoting manipulative or misleading information. Credibility tools provide quick assessments of trustworthiness to help analysts identify such outlets at the early stages of an investigation and to prioritise them for deeper scrutiny using additional methods.

In the context of TTPs, these tools support the detection of adversarial behaviours such as the exploitation of unverified sources, establishment of false fronts, and use of proxy outlets for amplification. Their role is therefore complementary to fact-checking tools: while fact-checkers verify the accuracy of individual claims, credibility assessment tools address the structural dimension of disinformation ecosystems, highlighting domains that systematically generate or disseminate manipulative content.

### 4.3 Bot detection tools

Bot detection tools address one of the most prominent techniques used in FIMI: the deployment of automated or semi-automated accounts to amplify narratives and simulate grassroots activity. RESONANT reviewed tools that apply behavioural analysis and machine learning techniques to distinguish between authentic user activity and automated or AI-generated content.

These tools typically operate by analysing features such as posting frequency, network connections, content similarity, and linguistic markers. By detecting anomalies in these patterns, they can identify accounts that contribute disproportionately to the visibility of specific narratives. In some cases, bot detection platforms also provide dashboards and scoring systems that allow investigators to monitor the scale and distribution of suspected inauthentic activity.

The relevance of bot detection tools to TTPs lies in their ability to expose adversarial behaviours such as coordinated amplification, astroturfing, and the artificial inflation of engagement metrics. By doing so, they help analysts identify when narratives are being promoted not through organic user interest but through orchestrated manipulation.

However, as noted in RESONANT, these tools often face limitations imposed by platform restrictions, particularly as social media companies tighten access to their data through application programming interfaces (APIs). While they provide valuable indicators, their effectiveness is dependent on the quality and availability of underlying data. Nonetheless, bot detection remains a crucial element in any toolkit designed to uncover the amplification phase of FIMI campaigns.

### 4.4 Image and video analysis tools

Image and video analysis tools constitute a broad category within the RESONANT review, reflecting the centrality of multimedia content in FIMI. Visual material is frequently used to anchor manipulative narratives, either by presenting fabricated evidence, altering authentic media, or repurposing content out of its original context. Tools in this category were therefore subdivided into four functional groups: image search, AI-generated image detection, image alteration detection, and optical character recognition (OCR).

**Image search tools** enable investigators to determine whether a visual asset has appeared elsewhere on the internet, thereby helping to establish provenance and detect reuse of content in misleading contexts. Such tools allow analysts to trace images back to their earliest known appearance, which is particularly relevant for identifying TTPs associated with content repurposing and strategic recycling of imagery.

**AI-generated image detection tools** respond to the increasing prevalence of synthetic media, such as deepfakes or artificially generated photographs. This tool category was reviewed in RESONANT for the tools’ ability to identify visual artefacts or statistical signatures typical of AI-generated content. Their role in TTP detection is linked to identifying fabrication techniques within the AMITT content creation phase, where adversaries employ synthetic visuals to enhance the credibility of disinformation campaigns.

**Image alteration detection tools** focus on identifying manipulations in authentic visual material, such as cropping, splicing, or retouching, and are capable of highlighting inconsistencies in compression, lighting, or metadata that may signal tampering. These tools help uncover TTPs associated with falsification and deceptive framing, where adversaries distort genuine evidence to support manipulative claims.

**Optical character recognition tools** extract embedded text from images or screenshots, enabling further analysis of content that would otherwise remain inaccessible to keyword searches. By converting visual text into machine-readable form, these tools facilitate both verification and content analysis, particularly when adversaries disseminate text-based disinformation in image formats to evade automated detection.

Taken together, image and video analysis tools form a vital component of the detection ecosystem. They provide investigators with the ability to interrogate visual material across multiple dimensions (provenance, authenticity, fabrication, and embedded content) thereby addressing adversarial procedures that exploit the persuasive power of multimedia.

### 4.5 Geo-IP verification tools

Geo-IP verification tools were included in RESONANT as a distinct category due to their role in linking digital traces to geographic locations. These tools provide analysts with information about the likely origin of an Internet Protocol (IP) address, often specifying country, region, city, and associated internet service provider. They enable investigators to conduct reverse lookups and determine the probable source of online activity.

The relevance of these tools for FIMI detection lies in their ability to connect online behaviours with physical infrastructure. Adversaries frequently rely on obfuscation techniques such as virtual private networks (VPNs) or proxy servers to conceal their location, yet patterns of IP use can still reveal clusters of activity or associations with particular geographic regions. Identifying these patterns contributes to the detection of adversarial TTPs, such as the use of anonymisation infrastructure or the coordination of campaigns across specific hubs.

From the perspective of technical attribution, Geo-IP tools provide supporting evidence that can be cross-referenced with other indicators. For example, repeated association of suspicious accounts with hosting infrastructure in particular regions may reinforce assessments of coordinated operations. Although such findings must be treated with caution, given the prevalence of obfuscation, they remain valuable for situating FIMI activity within a broader operational context. Thus, Geo-IP verification tools complement content- and network-focused detection methods by adding a geographic dimension to the analysis.

### 4.6 Phone-related tools

Phone-related tools were assessed in RESONANT as part of the broader detection ecosystem due to the recurring use of telephone numbers in disinformation campaigns and fraudulent online activity. Such numbers may be embedded in websites, social media posts, or messaging platforms as supposed points of contact, often intended to lend credibility to manipulative narratives or to facilitate direct engagement with targeted audiences.

These tools provide services such as carrier identification, reverse lookups and risk assessment. They can reveal whether a number is genuine, spoofed, or disposable and in some cases, offer insights into geographic origin or associations with known spam and fraud databases.

The contribution of phone-related tools to FIMI detection is relatively specialised but nonetheless, significant. They support the identification of adversarial TTPs linked to credibility-building through false contact information, fraudulent engagement tactics, and the use of disposable communication channels. Investigators can determine whether contact details are part of a broader manipulation strategy by verifying or discrediting phone numbers, thereby complementing other verification and attribution tools. While their relevance is narrower compared to categories such as social media analytics or multimedia forensics, phone-related tools address a specific detection need and contribute to building a comprehensive picture of adversarial operations. In doing so, they add another layer of evidence that can strengthen technical attribution when combined with other open-source indicators.

### 4.7 Social media analysis tools

Social media analysis tools were included in RESONANT as a distinct category due to the centrality of platforms such as Facebook, X (formerly Twitter), and Reddit in FIMI operations. These tools are designed to extract, filter, and monitor content from specific platforms, thereby providing insights into narrative dissemination, engagement dynamics, and potential signs of coordinated inauthentic behaviour.

From a TTP perspective, such tools are particularly relevant for detecting amplification and coordination techniques, since social media remains the primary arena for the spread of manipulative narratives. However, RESONANT also documented important limitations: many of the reviewed tools either returned incomplete or false results or were no longer fully functional due to platform restrictions on data access. This situation highlights a structural vulnerability in FIMI detection, where analysts are dependent on tools whose effectiveness can quickly diminish as platforms adjust their policies and technical environments. Despite these constraints, social media analysis tools remain essential for monitoring the real-time evolution of online campaigns, even if their long-term sustainability is uncertain.

### 4.8 Email verification tools

Email verification tools formed the final category examined in RESONANT. These tools are designed to validate the authenticity and deliverability of email addresses, detect the use of disposable or temporary domains, and in some cases perform reverse lookups to associate addresses with user profiles or organisations. The value of email verification tools for FIMI detection lies in their ability to expose adversarial TTPs that rely on fraudulent contact points or false identity markers. Manipulative campaigns often incorporate email addresses into websites, social media accounts, or outreach material as a means of lending credibility or facilitating direct interaction with targeted audiences. Investigators can quickly identify when such contact details are fabricated, high-risk or linked to disposable providers by verifying the status of these email addresses.

Similar to phone-related tools, their application is narrower compared to other categories such as multimedia analysis or social media monitoring. Email verification tools provide a complementary function within the overall detection process. They contribute to the broader objective of technical attribution by validating or discrediting contact vectors embedded within manipulative campaigns. In doing so, they add an additional evidentiary layer that, when combined with other open-source indicators, helps build a more comprehensive understanding of adversarial operations.

## 5. Results and analysis

The systematic testing of OSINT and verification tools within the RESONANT framework generated a wide range of empirical findings that shed light both on their technical performance and on their operational usability. The evaluation was conducted collaboratively by the cyberteams A (CT-A) and B (CT-B), consisting of Hellenic Police and Ukrainian Police experts, each testing the tools on distinct datasets under their respective operational conditions. While synthetic datasets were used to preserve ethical and legal safeguards, the results are representative of realistic FIMI detection scenarios and highlight the relative strengths and weaknesses of different tool categories. The comparative observations reported in this study reflect the specific testing period, tool versions, and dataset characteristics used during the controlled evaluation. OSINT tools evolve rapidly and their functionality, access conditions, and performance may change due to software updates, API modifications, or platform policy changes. Consequently, the results presented here should be interpreted as indicative of methodological applicability rather than as fixed performance rankings. Considering these remarks, in the following subsections, we provide an in-depth analysis of tool effectiveness and limitations, propose an optimised suite of instruments, and reflect on the practical challenges that arose during the testing and evaluation phases.

### 5.1 Tools’ effectiveness and limitations

The first category examined was **fact-checking tools**, which play a fundamental role in the early identification of false or recycled narratives. Across both evaluations, results showed clear disparities in performance. GPT-4o consistently outperformed all other tools, correctly identifying around 90 percent of true and 58 percent of false claims in the CT-A tests, and achieving 94 percent accuracy on true and 95 percent on false claims in the CT-B evaluation, with only minor false-positive rates. The Factual ranked second in the CT-A results, correctly classifying about 52–55 percent of true and 47 percent of false claims, though it showed higher uncertainty levels (up to 45 percent undefined). The CT-B results were broadly similar for true-claim detection, though other fact-checking repositories performed inconsistently on false claims. Traditional databases such as Snopes, Google Fact Check Explorer, PolitiFact, Truth or Fiction, AFP Fact Check, and Fake News Debunker provided only limited utility, often returning undefined outputs or failing to match phrasing with their existing entries. Collectively, the findings confirm that GPT-4o and The Factual currently represent the most reliable fact-checking instruments, while conventional repositories remain useful primarily for verifying widely circulating claims but lack the scalability and linguistic flexibility required for proactive FIMI detection.

The evaluation of **website-credibility assessment tools** further highlighted the uneven quality of existing resources. While the conceptual value of these tools lies in their ability to provide contextual judgments on the reliability of entire domains, their practical effectiveness was modest. NewsGuard, despite being widely cited in policy discussions, could not be tested due to its subscription-based model, and Schema.org’s ClaimReview lacked the necessary functionality for systematic evaluation. The Know News browser extension demonstrated very limited accuracy, correctly identifying credible or non-credible domains in only about 16 percent of cases. By contrast, MyWOT (Web of Trust), which was integrated after the main testing phase, proved more useful in assessing overall domain reputation. These findings illustrate a broader limitation of credibility-assessment approaches: although they can help highlight recurrent low-credibility sources, their coverage remains narrow and they often require human interpretation to validate results.

When evaluating **bot detection tools**, the testing process encountered major structural limitations. BotSentinel, once a widely used platform, discontinued its API service and was therefore unavailable for testing. GPTZero, designed primarily for detecting AI-generated text, was tested but could not be effectively applied to identifying automated social-media accounts. The limitation stemmed from the requirement to manually extract posts and comments from platform profiles—an action restricted under Twitter’s terms of service. These challenges highlight a broader issue in bot detection: effective analysis depends on access to authentic behavioral data, yet such access is increasingly limited by platform policies and API restrictions. Consequently, while bot detection remains an essential component of FIMI monitoring, current tools cannot be reliably deployed without sustainable and compliant data-access mechanisms.

The **image and video analysis** category showed strong and consistent performance, making it one of the most reliable layers of the toolkit. **Reverse-image-search tools**, including *Google Images*, *TinEye*, and *Search by Image*, achieved 100 percent accuracy, successfully identifying all instances of manipulated or recycled images within the dataset. Tools designed to detect AI-generated content produced more mixed results: *ContentAtScale.ai* and the *Umm-maybe* model hosted on Hugging Face correctly identified about 72 percent of real images and 52–56 percent of AI-generated ones, while *Illuminarty.ai* performed poorly, detecting only around 20 percent of fake images and generating a high number of false positives. **Image-alteration-detection tools**, such as *FotoForensics* and *29a.ch Photo Forensics*, also showed uneven but useful performance, both detected 100 percent of genuine images, while achieving 60 percent and 40 percent accuracy, respectively, on altered ones, yet their results required expert interpretation. **OCR** tools performed very strongly: *Google Lens* achieved 92 percent accuracy, *ChatGPT OCR* 88 percent, and *OCR.space* 68 percent, clearly outperforming older tools such as *NewOCR.com* (28 percent) and *OnlineOCR.net* (16 percent). Taken together, these results indicate that while multimedia forensics is not immune to errors, the current ecosystem of tools for reverse search, AI-image detection, alteration analysis, and text extraction is relatively mature and can be effectively operationalised for investigative use.

The **infrastructure-level tools** produced more varied results. **Geo-IP detection platforms** displayed a moderate degree of accuracy. *MaxMind GeoIP* failed to operate during testing, while *IPFingerprints* achieved only 36 percent accuracy in identifying the correct country. By contrast, *IPLocation.net* and *Thatsthem IP Lookup* reached about 70 percent accuracy, indicating that while IP geolocation cannot serve as a standalone attribution method, it provides a useful supporting layer for situating suspicious activity within broader geographic patterns. **Phone-number-verification tools** showed clearer contrasts: *WhoCalled.com* delivered the strongest results, correctly matching approximately 89 percent of tested numbers in both evaluations and, in several cases, even identifying partial owner information. *IPQualityScore’s* validator achieved about 65 percent accuracy in the CT-A results but did not return usable data in the CT-B evaluation, as it required manual entry of country codes for each query. *Truecaller*, despite its popularity, lacked the necessary functionality in its free version and could not be properly tested without login credentials. Overall, the results confirm that *WhoCalled.com* offers the highest reliability for phone-number analysis, while other tools remain constrained by technical or access limitations.

Finally, **social-media-analysis tools** proved to be the least reliable category. Many of the platforms previously used for extracting or monitoring social-media data, such as *CrowdTangle*, *Ad Observer*, and *SearchIsBack*, are now deprecated, restricted, or entirely non-functional. Tools designed for *Twitter*, including *Advanced Search* and *Geo-Search *, often returned incomplete or false results, while *Facebook*-based searches and numerous *Reddit* utilities such as *SubredditStats*, *Reddit User Analyser*, and *Apify Reddit Scraper* either failed to respond or produced accuracy levels below 40 percent. Several others, including *Redective*, *Keyworddit*, and *SowSearch*, produced errors or unreliable outputs. Overall, the category was marked by discontinued services, false data, and API restrictions imposed by major platforms. This reflects a broader structural issue: as social networks tighten data access, third-party analytical tools rapidly become obsolete. Consequently, while social-media analysis remains essential for FIMI detection, the current landscape is unstable and requires renewed development of sustainable, API-driven, and institutionally supported solutions.

The evaluation of **email-verification tools** produced mixed but informative results. Among the tools tested, *MailboxValidator* and *Thatsthem Reverse Email Lookup* delivered the most consistent performance, correctly identifying around 21–22 percent of valid addresses and providing detailed analytical parameters. *EmailHippo* produced useful outputs but showed a higher rate of unverifiable results (18 percent) and lower overall accuracy. *Emailable* and *EmailValidator.com* could not be tested effectively due to account or subscription restrictions. In the CT-B tests, *MailboxValidator* again demonstrated stable functionality, while *Thatsthem* provided only partial informational outputs without explicit verification results. Overall, the findings indicate that while several email-verification tools remain limited by access or functionality constraints, *MailboxValidator* and *Thatsthem* together offer the most reliable combination for validating contact information within OSINT investigations.

**Integrated and cross-domain evaluations**

Beyond single-function testing, a series of integrated evaluations was conducted to measure interoperability and cross-domain performance. In the **Comprehensive Media Analysis**, the CT-A achieved strong, domain-specific results across all stages: *29a.ch Photo Forensics* reached 100 percent accuracy in image and video analysis, *Originality.ai* achieved 89–90 percent accuracy in content verification, and *EmailHippo* succeeded in all contact-validation tests. CT-B obtained comparable but more qualitative outcomes: *29a.ch Photo Forensics* effectively detected image alterations such as cloning but provided limited contextual detail; *Truecaller* functioned well as a mobile application for identifying spam or malicious phone numbers, and *The Factual* produced a “Moderately Credible” score of 51 percent for article reliability, performing effectively on most standard-length articles. Taken together, these findings demonstrate that well-configured combinations of content, media, and contact-verification tools can deliver near-complete analytical coverage, though interpretation still requires a degree of manual expertise.

The **Disinformation Campaign Analysis** revealed mixed levels of effectiveness across the tested tools. For CT-A, *The Factual* proved highly reliable, achieving 100 percent success in article analysis, while *Search by Image* reached 43 percent accuracy in image and video verification, and *Know News* scored 0 percent in social-interaction analysis. These results highlight the strong performance of text-based verification tools but also the need to integrate more robust image-analysis solutions and alternative methods for assessing online interactions. CT-B obtained generally effective outcomes, particularly for article analysis, with *Know News* performing well in credibility assessment and *FotoForensics* offering detailed image insights that nevertheless required user expertise to interpret accurately. Overall, the findings confirm that while content-verification tools are mature and reliable, image forensics and social-interaction mapping remain dependent on operator skill and would benefit from more advanced, automated capabilities.

The **Cross-Platform Disinformation Analysis** produced mixed results. In CT-A’s evaluation, *The Factual* recorded 0 percent success in article verification, while *MediaBiasFactCheck* achieved 100 percent accuracy in social-media analysis. *CrossRef Search* failed to generate relevant matches (0 percent), and *MonkeyLearn Sentiment Analysis* produced a 40 percent positive versus 60 percent negative sentiment balance. CT-B reported more stable outcomes, with *The Factual* performing effectively in assessing article reliability. Overall, the findings indicate that while textual and sentiment-analysis tools can yield valuable insights, the ability to establish reliable cross-platform correlations remains limited, highlighting the need for better-integrated analytical frameworks within the current OSINT ecosystem.

Finally, the **Multi-Source Disinformation Campaign Analysis** assessed the toolkit’s capacity to detect and contextualize disinformation across complex geopolitical narratives. In CT-A’s assessment, *The Factual* achieved 42 percent success in article analysis, *Scribbr AI Detector* reached 14 percent in social-media analysis, and *X.com* *Explore* delivered 100 percent accuracy in cross-reference detection. CT-B did not report available data for this phase. Overall, the results indicate that while content-verification tools can provide partial success in large-scale misinformation environments, automation and cross-modal integration remain limited, underscoring the continued need for human oversight and contextual reasoning in comprehensive disinformation analysis.

**OSINT tools: Limitations**

Although OSINT tools often seem to be very powerful while conducting digital investigations, due to the automating and fast way of functioning, still they present some significant limitations.

One major limitation is their volatility. In general, digital platforms, social networks, and online repositories constantly change their structures, restrict access, or modify interfaces, depending on various administrative, legal and other reasons. Of course, this also has an impact on an OSINT tool, as these changes may most likely prevent it from functioning. Thus, this constantly unstable situation should be taken into consideration by all interested persons/organizations, who should not rely heavily on any single automated solution.

Next, another limitation is the narrow scope of data coverage. For example, an OSINT tool may be designed to focus on a specific category, such as IP addresses, domain records, usernames, or social media accounts, while leaving many others uncovered, of high importance. This can lead to the conclusion that no single tool can ever provide a complete intelligence picture, and important information can easily be missed if investigations rely solely on tool-generated results.

Another limitation of OSINT tools is the reliability of the generated data. Many tools rely on old, outdated or limited/incomplete sources, which often results in misleading and inaccurate findings. For this reason, it is vital to properly validate and cross-check all resulting information prior to its use or dissemination.

Furthermore, a common risk that someone can fall into is the lack of security measures. This risk emerges from the fact that many OSINT tools interact directly with target platforms (domains), leaving residual evidence in the target log files. This could potentially raise an alert for the administrator, indicating that an investigation is underway. For this reason, proper operational security practices, such as anonymisation, virtual environments, and sock puppets are essential to avoid these risks.

The previous point is reinforced by the fact that there is often an “automation mentality” that gives the impression that OSINT tools are simple, requiring only pressing a button and wait for the results. This creates a false sense of security as, for sure, the tools can assist in the intelligence gathering process, but they cannot replace human creativity, analytical thinking, and reasoning.

Given the rapid evolution of OSINT platforms and access conditions, repeated testing over time would likely produce different operational outcomes.

In summary, OSINT tools are valuable for increasing speed and efficiency, but they have clear limitations. They cannot ensure complete coverage, absolute accuracy, or replace human judgment. Successful OSINT work relies on the investigator’s skills, operational security, and solid methodology, with tools acting as supporting resources rather than definitive solutions.

### 5.2 Optimised tool suite

On the basis of the comparative evaluation, an optimised suite of tools was proposed for integration into the RESONANT detection environment. At the content-verification level, GPT-4o and The Factual were retained as the most accurate and scalable fact-checking instruments, with traditional repositories maintained in reserve for redundancy and historical coverage. Originality.ai was included as a complementary resource for automated content verification. For website credibility, MyWOT was prioritized as the most consistently usable tool, with Know News included as a secondary option. In the domain of multimedia forensics, Google Images, TinEye, and Search by Image were selected for reverse search; ContentAtScale.ai and the Umm-maybe model (Hugging Face) were retained for AI-image detection; FotoForensics and 29a.ch Photo Forensics were integrated for alteration analysis; and Google Lens and ChatGPT OCR were identified as the strongest performers for text extraction. At the infrastructure level, IPLocation.net and Thatsthem IP Lookup were recommended for IP geolocation, WhoCalled.com for phone verification, and a combination of MailboxValidator and Thatsthem Reverse Email Lookup for email verification.

Although bot detection and social-media analytics remain structurally important, the evaluation concluded that current tools are either deprecated or insufficiently reliable, underscoring the need for further development and integration of sustainable, API-driven solutions. The same applies to cross-platform and multi-source disinformation analysis, where tool interoperability and automation are still limited.

This optimised suite reflects a layered and complementary approach to tool selection. No single tool or category was sufficient to address the full spectrum of adversarial tactics, techniques, and procedures. However, by combining high-performing instruments across content, platform, and infrastructure levels, it is possible to build a detection ecosystem that compensates for individual weaknesses. The proposed suite is therefore not a static configuration, but a dynamic baseline, designed to evolve as tools are updated, discontinued, or replaced.

### 5.3 Practical challenges

In addition to technical performance, the evaluation process exposed several practical challenges that must be considered in the operationalisation of OSINT methodologies. The first challenge relates to **sustainability**: many tools tested were found to be discontinued, inaccessible, or locked behind subscription models, sometimes in the middle of the evaluation itself. This instability undermines the reproducibility of results and highlights the need for a more institutionalized approach to tool provision, potentially through public-private partnerships or open-source development.

A second challenge concerns **interoperability**. While the testing aligned tool outputs with structured frameworks such as DISARM and AMITT, few tools natively export results in machine-readable or standardized formats. This lack of integration increases the burden on analysts and limits the scalability of detection efforts. Without interoperability, it is difficult to link outputs across categories or feed them into broader intelligence platforms.

Third, the evaluation revealed persistent **coverage gaps**, particularly regarding multilingual detection and non-Western information environments. Most fact-checking repositories are heavily biased towards English-language content, while many credibility tools assess primarily Western news outlets. This creates blind spots that adversaries can exploit by targeting under-represented linguistic or regional contexts.

Significant ethical and legal constraints arise in the use of OSINT tools, particularly concerning bot detection and the scraping of data from social media platforms. These challenges are especially relevant in operational settings, where strict compliance with data protection regulations (such as the GDPR) and adherence to platform terms of service are required. While synthetic datasets can help mitigate some of these risks during development and testing, they do not eliminate the legal and ethical complexities involved in real-world applications. As emphasised in the attribution literature, technical observables identified through OSINT tools can support investigations but cannot, on their own, establish responsibility for information influence operations, which ultimately requires contextual, political, and legal assessment. ^31^ Operational usability is another critical factor that can affect the effectiveness of OSINT tools. Even when tools are technically accurate, their practical value may be reduced by unclear user interfaces, non-transparent scoring systems, or steep learning curves. These issues can limit accessibility and hinder adoption by investigators and analysts. Beyond legal compliance, ethical considerations play a vital role in the responsible use of OSINT. Analysts must ensure that their actions are necessary, proportionate and respectful of fundamental rights. This includes avoiding techniques that could be seen as intrusive, manipulative, or misleading. In the absence of universally accepted international standards, OSINT practitioners must rely on sound professional judgment and uphold principles of transparency, accountability and integrity in their work.

Taken together, these challenges highlight the need for a robust, flexible, and ethically grounded framework for OSINT testing and validation. The RESONANT methodology provides an initial foundation by consolidating tool evaluations and aligning them with structured frameworks. However, further efforts will be required to ensure sustainability, interoperability, and accountability in the rapidly evolving ecosystem of FIMI detection tools.

## 6. Conclusion and future research

This article has presented a structured methodological framework for testing OSINT tools in the detection of FIMI-linked operations. Building on established taxonomies such as DISARM, AMITT, and MITRE ATT&CK, the framework consolidates conceptual, operational, and evaluative elements into a coherent process. Its primary contribution lies in bridging a gap frequently noted in the literature: while numerous studies analyse OSINT techniques conceptually, few provide empirically grounded, reproducible procedures for systematically evaluating FIMI-related detection tools. This framework offers a transparent and transferable model that advances methodological rigour in the OSINT domain through practitioner input, synthetic datasets and controlled testing conditions integration.

A key contribution is the articulation of a multi-layered detection logic, distinguishing content-level verification, platform-level activity analysis and infrastructure-level attribution. Previous research highlights the importance of these layers, yet assessments often remain fragmented, focusing on individual tools or isolated use cases. The RESONANT methodology operationalises this layered understanding, demonstrating how complementary strengths across categories can mitigate inherent weaknesses. This offers researchers and law-enforcement agencies a more coherent lens through which to interpret the ecosystem of FIMI detection tools.

The study also contributes to the field by showing the value of synthetic, ethically compliant datasets for investigating adversarial behaviours without exposing sensitive or personal information. While synthetic data are used in OSINT research, they are rarely embedded into a full evaluation pipeline with reproducibility and traceability safeguards. The controlled testing environment developed here allows tools to be compared under identical conditions, which is an essential step for performance-based decisions in operational settings. This represents an advancement over prior approaches that often rely on ad hoc testing or real-world data whose provenance, privacy, or representativeness may be uncertain.

At the same time, the study highlights several limitations that inform directions for future work. First, reliance on synthetic datasets (while necessary for ethical and security reasons) cannot fully capture the dynamic unpredictability of evolving information manipulation tactics. Second, OSINT tools operate within a rapidly changing digital landscape: API access restrictions, platform policies, and data availability constraints can affect tool performance over time. Continuous re-validation and updating of the dataset design and testing environment will therefore be essential. Third, the methodology focuses on detection rather than classification of intent or attribution, areas that require further analytical and legal input.

Looking forward, the proposed framework can support a number of research and operational pathways. Future studies could expand the testing protocol to integrate machine-learning-driven tools, automated pipeline orchestration and cross-platform detection capabilities. There is also scope to explore interoperability with threat-intelligence platforms and harmonisation with EU-level standards for analysing information manipulation activities. Finally, applying the methodology across additional languages, geopolitical contexts, and sociotechnical environments would enhance its generalisability and support comparative research across the FIMI domain.

In sum, this study contributes to strengthening the evidence base for OSINT tool evaluation in the context of FIMI detection. By offering a reproducible, ethically grounded, and operationally informed framework, it advances both scholarly understanding and practical capabilities for countering information manipulation, supporting broader EU efforts to enhance democratic resilience and information security.

## Ethics and consent

Ethical approval and consent were not required for this study. The research did not involve human participants, personal data, or real-world operational datasets. All testing activities were conducted using synthetic datasets developed within the RESONANT project exclusively for methodological validation purposes.

## Use of generative AI tools

The authors used ChatGPT (OpenAI) to support language refinement, structuring of sections, and editorial improvements. The tool was not used to generate original scientific content, interpret results, or draw conclusions. All content was reviewed and approved by the authors.

## Data Availability

Zenodo. *Detecting FIMI: A methodological framework for testing OSINT tools in TTPs detection.* https://doi.org/10.5281/zenodo.19045753. ^32^
• The Zenodo record contains supporting methodological materials associated with the study, including documentation of the testing framework and related non-sensitive materials. Data are available under the terms of the Creative Commons Attribution 4.0 International (CC BY 4.0) license. The research presented in this article did not involve personal, operational, or otherwise sensitive real-world data. All testing activities were conducted using synthetic datasets created within the Horizon Europe project RESONANT (Grant Agreement No. 101132439). These datasets were specifically designed to simulate FIMI scenarios, including fabricated narratives, manipulated images, simulated bot activity, and spoofed infrastructure elements. The full synthetic scenarios, detailed dataset structures, and related testing materials are not publicly available due to security considerations. Although these datasets do not contain personal data, their structure could potentially be misused to replicate manipulation techniques or adversarial behaviours. No Institutional Review Board approval was required, as the study did not involve human participants or personal data. The restrictions on data sharing were assessed internally in accordance with the RESONANT Data Management Plan and Data Protection Impact Assessment (DPIA), which concluded that controlled access was necessary to mitigate misuse risks. Access to the restricted materials may be granted to bona fide researchers for scientific verification purposes upon reasonable request, subject to a justified research purpose, agreement to conditions of use including non-disclosure and non-operational reuse, and approval by the RESONANT consortium. Requests should be addressed to the corresponding author at the Center for Security Studies (KEMEA), k.margaros@kemea-research.gr Information on the OSINT tools analysed in this study was collected through a structured multi-source approach drawing on publicly available materials, academic and grey literature, EU-funded project outputs, repositories such as AMITT, and the operational expertise of RESONANT partners.

## References

[1] EEAS: *2022 report on EEAS activities to counter FIMI.* European External Action Service;2023. Reference Source

[2] EEAS: *How to Detect & Analyse Identity-Based Disinformation/FIMI: A Practical Guide to Conduct Open Source Investigations.* European External Action Service;2024. Reference Source

[3] Government of Canada: *Foreign information manipulation and interference targeting Canada’s democratic processes: Special report for 2019–2023.* Canadian Security Intelligence Service (CSIS) & Communications Security Establishment (CSE);2023.

[4] CogSec Collaborative, & Threet LLC: AMITT TTP guide. n.d. Reference Source

[5] Dou W Wang X Ribarsky W : *Event detection in social media data.* University of North Carolina at Charlotte & IBM Almaden Research Center;2012.

[6] Chinnov A Kerschke P Meske C : An overview of topic discovery in Twitter communication through social media analytics. In Proceedings of the Twenty-first Americas Conference on Information Systems (AMCIS 2015). 2015.

[7] Ferrara E Varol O Davis C : The rise of social bots. *Commun. ACM.* 2016;59(7):96–104. 10.1145/2818717

[8] Demetzou K : Data protection impact assessment: A tool for accountability. *Computer Law & Security Review.* 2019;35(6):105339. 10.1016/j.clsr.2019.04.003

[9] Sabir MI : Assessing attribution and credible deterrence in cyberspace. *J. Cyber Policy.* 2024;9(1):44–63. 10.1080/23738871.2024.2301346

[10] Tsagourias N Farrell C : Cyber attribution: Technical and legal approaches and challenges. *Research handbook on international law and cyberspace.* Edward Elgar Publishing;2020; pp.212–229. 10.4337/9781789904255.00018

[11] CogSec Collaborative, & Threet LLC: AMITT user guide. n.d. Reference Source

[12] CogSec Collaborative, & Threet LLC: AMITT incident list. n.d. Reference Source

[13] Rani N Bansal A Batra S : A comprehensive survey on advanced persistent threat attribution with enabling paradigms and challenges. *J. Netw. Comput. Appl.* 2024;239:103738. 10.1016/j.jnca.2024.103738

[14] Aral S Dellarocas C Godes D : Social media and business transformation: A framework for research. *Inf. Syst. Res.* 2013;24(1):3–13. 10.1287/isre.1120.0470

[15] Kane GC Alavi M Labianca GJ : What’s different about social media networks? A framework and research agenda. *MIS Q.* 2014;38(1):275–303. 10.25300/MISQ/2014/38.1.13

[16] Qin Z Zhou Y Wu T : How rumors spread and stop over social media: A multi-layered communication model. *HCIS.* 2015;5(1):1–21. 10.1186/s13673-015-0027-1

[17] Mattingly D Yao E : How soft propaganda persuades. *Comp. Pol. Stud.* 2022;55(14):2427–2455. 10.1177/00104140221119176

[18] Dokman T Ivanjko T : Open source intelligence (OSINT): Issues and trends. *INFuture 2019: Knowledge in the digital age.* University of Zagreb;2019; pp.191–196. 10.17234/INFUTURE.2019.23

[19] Hwang J Kim S Lee J : Current status and security trend of OSINT. *Journal of Information and Security.* 2022;2022(4):345–360. 10.1155/2022/1290129

[20] Tuominen S : OSINT: Intelligence and its application. *Poliisiammattikorkeakoulun raportteja.* 2019;138:1–74. Reference Source

[21] Glassman M Kang M : Intelligence in the internet age: The emergence and evolution of Open Source Intelligence (OSINT). *Comput. Hum. Behav.* 2011;28(2):673–682. 10.1016/j.chb.2011.11.014

[22] Nobili M : A systematic review of OSINT tools for social engineering. *J. Digit. Forensic Secur. Law.* 2023;18(1):1–20. 10.15394/jdfsl.2023.1865

[23] Julan CI Togan M : Methodologies for retrieving and processing information from open sources (OSINT). *Journal of Military Technology.* 2023;6(1):39–44. 10.32754/JMT.2023.1.05

[24] Hassan N Hijazi R : *Open source intelligence methods and tools: A practical guide to online intelligence.* Apress;2018. 10.1007/978-1-4842-3213-7

[25] Meredith A : *The OSINT handbook: A practical guide to intelligence gathering.* Routledge;2024.

[26] Adetokunbo A Awodele O Ogbonna AC : A review of the waterfall model and object-oriented approach. *Int. J. Eng. Res. Appl.* 2013;3(6):729–732.

[27] Andrei B : A study on using waterfall and agile methods in software project management. *Journal of Engineering Studies and Research.* 2019;25(3):106–111. 10.29081/jesr.v25i3.0000

[28] Aroral H : Waterfall process operations in the fast-paced world. *International Journal of Creative Research Thoughts.* 2021;9(6):2324–2330.

[29] Senarath U : Waterfall methodology, prototyping and agile development: A comparative study. *Int. J. Adv. Res. Comput. Sci.* 2021;12(3):1–7. 10.26483/ijarcs

[30] Eason O : An analysis of waterfall to agile methodology. *International Journal of Engineering Research and General Science.* 2016;4(3):781–788.

[31] Pamment J Smith V : *Attributing Information Influence Operations: Identifying those Responsible for Malicious Behaviour Online.* NATO Strategic Communication Centre of Excellence;2022. Reference Source

[32] Margaros K Paparrigopoulos T Filippopoulos O : Detecting FIMI: A methodological framework for testing OSINT tools in TTPs detection.[Data set]. *Zenodo.* 2026. 10.5281/zenodo.19045753

