# Supplementary material for: Detecting FIMI: A methodological framework for testing OSINT tools in TTPs detection
Source: Open Res Eur. 2026 Aug 8;6:157. Originally published 2026 May 9. [Version 2] doi: 10.12688/openreseurope.21702.2 (PMC13560597; doi:10.12688/openreseurope.21702.2)
Supplement: Peer review history

# Reviewer report: Reviewer response for version 2

**DOI:** 10.21956/openreseurope.26801.r78801

**Published:** 2026-08-26

**Authors:** Marius Dragomir

**Affiliations:** 1 University of Santiago de Compostela, Santiago de Compostela, Spain

No more comments to make.

Is the rationale for developing the new method (or application) clearly explained?

Yes

Is the description of the method technically sound?

Partly

Are the conclusions about the method and its performance adequately supported by the findings presented in the article?

Partly

If any results are presented, are all the source data underlying the results available to ensure full reproducibility?

Partly

Are sufficient details provided to allow replication of the method development and its use by others?

No

Reviewer Expertise:

Disinformation, Media literacy, Media Studies, Broadcast regulation, PSM, Audience studies, Media sustainability,

I confirm that I have read this submission and believe that I have an appropriate level of expertise to confirm that it is of an acceptable scientific standard.

# Reviewer report: Reviewer response for version 2

**DOI:** 10.21956/openreseurope.26801.r78802

**Published:** 2026-08-20

**Authors:** Giovanni Spitale

**Affiliations:** 1 IBME, Universitat Zurich, Zurich, Switzerland

This is a serious revision. The authors have engaged with all eight major comments from my first report, and in several places they have gone beyond the minimum: the systematic-review framing has been withdrawn, the evaluation corpus is now described accurately as mixed-source rather than synthetic, the performance figures have been demoted from rankings to time-bound descriptive observations, and the ethics-by-design claim has been downgraded to a guiding orientation rather than defended as a measured outcome. That last move is uncommon in revision and it improves the paper: where weakening a claim was the honest option, the authors weakened it. Appreciated. The new material in par 4.1 on content-dependent versus content-agnostic detection, the bias paragraph and operational safeguards in par 5.2, the decision protocol in par 5.2, the legal-versus-epistemic-ethics distinction in par 5.3, and the regulatory-access paragraph in par 6 address the substance of what I raised rather than gesturing at it.

 My principal objection to version 1 was that the central empirical claim was not interpretable as stated and that the normative framework addressed lawfulness only. Both have been fixed properly rather than cosmetically, and the claims now match the evidence that supports them. I therefore move from Not Approved to Approved with Reservations.

 The reservations that remain are of two kinds. Three concern the material deposited for this revision, which I checked against the article, and which does not currently do the work the article claims for it. The remainder concern residual sentences where the revised framing has not been propagated to text surviving from version 1. None require new data collection or re-analysis.

**Disposition of the Version 1 comments**

**MaC1(a), circularity of the evaluation corpus for LLM testing: resolved.** The first caveat in par 5.1 states the training-data recognition problem directly, par 3.2 repeats it, and the fact-checking paragraph now closes with the inference I said was missing, namely that the figures "should not be read as evidence that GPT-4o is the most reliable operational fact-checking instrument for novel, emerging, or out-of-distribution FIMI content". The study was not re-run on out-of-distribution material, but the paper no longer claims what such a re-run would be needed to support.

**MaC1(b), inter-rater reliability: addressed by disclosure rather than by measurement.** No coefficient was computed, and par 3.2, par 3.4, the fourth caveat in par 5.1 and the deposited README all say so. For a historical dataset this is acceptable, provided the consequence is stated, and par 5.1 now states it: the percentages are agreement with project-defined labels of unvalidated quality, not externally validated accuracy.

**MaC1(c), the CT-A/CT-B divergence: resolved.** The third caveat in par 5.1 names the 58 versus 95 percent gap explicitly and attributes it to the design rather than to the tool.

**MaC1(d), the comparison as a validation strategy: largely resolved, with one gap.** The clarification added at the end of par 3.3 is exactly right. It is not, however, applied at the point of use. The "Integrated and cross-domain evaluations" subsection still presents the most extreme cross-team contrasts in a comparative register, notably The Factual at "100 percent success in article analysis" for CT-A in the Disinformation Campaign Analysis and "0 percent success in article verification" for CT-A in the Cross-Platform analysis, set against CT-B's "more stable outcomes", with no reminder that these are not comparable quantities. One sentence at the head of that subsection referring back to par 3.3 would close this.

**MaC2, systematic bias of LLMs on evaluative tasks: resolved.** The paragraph added to par 5.2 is well judged. It names source-framing effects and broader ideological bias, correctly locates the mechanism in training-data and alignment regularities rather than in prompting, observes that GPT-4o is the least transparent instrument in the proposed suite, and makes its inclusion conditional on presenting content without source attribution and cross-validating against source-agnostic tools.

**MaC3, legal compliance versus epistemic ethics: resolved.** The new paragraph in par 5.3 draws the distinction and, importantly, connects it to the protocol in par 5.2 rather than leaving it as a standalone observation.

**MaC4, the content-level detection paradigm: resolved.** The closing paragraph of par 4.1 theorises the limitation as structural rather than contingent and distinguishes content-dependent from content-agnostic approaches. See MiC3 for the one place where the conditioning is promised but not applied.

**MaC5, normative architecture of the suite: resolved.** The three-point protocol in par 5.2 is minimal but real, and it does the required work: verdict outputs as leads rather than conclusions, independent corroboration before investigative action, human override on divergence.

**MaC6, the misinformation/disinformation conflation: partly resolved.** The terminological paragraph in par 1 is clear and correct. It also makes a promise: "Where relevant, the analysis therefore distinguishes which layer of the tool suite addresses which type of epistemic harm". Sections 4 and 5 do not deliver this; the distinction is drawn once and then not used. Either deliver it, for which a clause per layer in par 5.2 would suffice, or soften the promise in par 1.

**MaC7, the ethics-by-design claim: resolved.** The second paragraph of par 3.5 is the honest version, and identifying the operationalisation of bias, transparency and explainability metrics as a priority for future iterations is the right disposition.

**MaC8, the regulatory gap in future directions: resolved.** The penultimate paragraph of par 6 names the question and identifies public-interest or institutionally guaranteed access to platform data as the most pressing open problem for the field.

**Major Comments**

**MaC9: The Data availability section links to the superseded version of the Zenodo record (Data availability)**

 "Zenodo. Detecting FIMI: A methodological framework for testing OSINT tools in TTPs detection. https://doi.org/10.5281/zenodo.19045753"

 That DOI is the version-specific identifier for version 1 of the deposit, published 16 March 2026, which contains two files: "Resonant documentation file.xlsx" (14.6 kB) and "Resonant Metrics.pdf" (601.6 kB). It is precisely the deposit that the Amendments note describes as insufficient: "the previous deposit contained only the metrics document and the documentation template". A reader following the citation arrives at a page headed "There is a newer version of the record available" and finds none of the corpus the article describes in Data availability.

 The corpus was in fact deposited, as version 2.0, published 27 July 2026, 60.5 MB, comprising README.md, 1-4.zip, 5-8.zip, 9-12.zip and the two original documentation files. I verified its contents against the inventory given in the article and they correspond. The problem is one of citation only, and is trivially fixable, but until it is fixed the central transparency claim of this revision cannot be checked by any reader who follows the reference. Given that the deposit is what converts several of the version 1 objections from unresolved to resolved, this is not a cosmetic error.

**RECOMMENDED FIX:** cite the concept DOI 10.5281/zenodo.19045752, which always resolves to the latest version, or the version DOI 10.5281/zenodo.21627200. The same correction is required in Section 6 of the deposited README, which also cites the version 1 DOI. Two related items on the Zenodo side: the version 2.0 record's "Is supplement to" relation still points to 10.12688/openreseurope.21702.1 rather than to the current article version, and the record Description still lists only the template and the metrics document, with the corpus described in a separate Methods field, so a reader browsing the record sees the version 1 description attached to the version 2 files.

**MaC10: The deposited README carries unexecuted pre-publication instructions, and provenance for the largest components is unconfirmed (Data availability, par 3.5, par 5.3)**

 The README is otherwise a good document, considerably better than what most method papers deposit, which is what makes this visible. Its file inventory carries repeated editorial notes addressed to the authors rather than to users: "Confirm original source and licence." against fake.txt and real.txt; "Confirm source; treat as synthetic/illustrative unless otherwise stated." against bot_detection_data.csv; "Confirm source and redistribution licence." against asn-ipv4.csv and country_asn.csv. Section 5 then states: "Before publishing, the authors should confirm and record the exact source and licence for: the fake/real text corpora, the bot-detection dataset, the IP→ASN reference tables, the email corpus, and the website list."

 That instruction was not carried out before the record was published. Three consequences follow, in ascending order of seriousness.

 Epistemically, the unconfirmed provenance prevents a reader from evaluating the contamination caveat the authors added in response to MaC1(a). That caveat holds that GPT-4o "may achieve high apparent accuracy by recognising content encountered during training". Whether this risk is near-certain or negligible depends entirely on the origin of the 12,228 fake-labelled and 9,762 real-labelled text items. If they derive from one of the widely mirrored public fake/real news collections, contamination is close to guaranteed; if the material is project-generated, it is not. The README does not say, so the caveat cannot be assessed by the reader for whom it was written. Naming the source resolves this at no cost and would strengthen the caveat rather than weaken it.

 Legally, the record is released under CC-BY 4.0 while redistributing components whose licences and database rights are, on the authors' own account, not established. The record's copyright note that third-party data is not re-licensed, and the README's assignment of compliance responsibility to re-users, mitigate this but do not resolve it: responsibility for observing a licence cannot be transferred for an act of redistribution that may not have been permissible in the first place.

 Ethically and in data-protection terms, this is the point that bears most directly on the article's own commitments. The deposit redistributes email/*.eml, described in the README as 100 files with "real message headers", from a source recorded as a "third-party/public email corpus" with the licence unconfirmed, together with PhoneBookDataset.csv containing 441 rows of real contact data. An article whose par 3.5 invokes the project DPIA, purpose limitation and data minimisation, and whose par 5.3 argues that "public availability does not remove the need for appropriate handling", should not publish personal data whose lawful basis for onward publication is marked as pending in its own documentation. The README states the correct principle and the deposit does not yet meet it.

 Recommended fix, in order of preference: confirm and record the sources and licences as the README itself instructs; where a source cannot be established, withdraw that component from the deposit and note the withdrawal in the README; and consider whether the email and phone-contact files need to be redistributed at all, since none of the article's claims depend on a reader possessing them. On any route, the "Confirm..." annotations should not remain in the published README.

**MaC11: The instrument identified in the deposited baseline is not the one the article names (par 5.1, par 5.2)**

 The article refers throughout to GPT-4o. The deposited baseline document, Resonant Metrics.pdf, p. 3, lists the tool tested in the fact-checking category as https://chatgpt.com, and lists ChatGPT OCR separately in the multimedia category. If the testing was conducted through the consumer chat interface rather than through the API, two consequences follow that bear on interpretation.

 First, the evaluand is not the GPT-4o model but a product surface whose behaviour depends on the served snapshot, the system prompt and whether browsing and tool use were active, none of which the tester controls or observes. Second, if browsing was available, part of the reported accuracy may reflect retrieval of published fact-checks at query time rather than model knowledge or independent verification. That is a mechanism distinct from the training-data recognition addressed in the first caveat of par 5.1, and it changes what the figures mean: a retrieval-assisted interface evaluated on documented cases behaves more like a search tool than like a classifier, and the resulting numbers say little about performance on content for which no published fact-check exists.

 The article already concedes that "the exact historical GPT-4o snapshot, prompt, temperature and tool/browsing configuration were not retained". The present request is narrower and should be answerable from the testing partners' recollection: state which interface was used, and whether browsing was enabled, disabled, or not known. If the web interface was used, the instrument should be named accordingly throughout, for instance as ChatGPT (GPT-4o era, web interface), rather than as the model. This also affects par 5.2, where the recommendation to retain the tool in the operational suite is a recommendation about a specific access mode with specific safeguards attached.

**Minor Comments**

**MiC1: The System Usability Scale statement contradicts the deposited documentation (par 3.4)**

 Par 3.4 states that "The retained records do not document a consistently administered System Usability Scale". Resonant Metrics.pdf, p. 8, states that "The System Usability Scale (SUS) was used to measure ease of use/user experience", lists three adapted items, and specifies that "Each team compiled only one response per tool based on its use in the various scenarios." Both statements can be true of the same facts, since a three-item adaptation is not the ten-item instrument, but as written the article and its own deposit contradict each other on their face. One clarifying sentence would resolve it, and should carry the detail that matters more than the label: usability was recorded as a single consolidated response per team per tool. That is a defensible design for an operational evaluation, but it bounds what "practitioner validation" can be taken to mean, and readers should be told in the article rather than left to discover it in the appendix.

**MiC2: Percentages are reported without denominators anywhere in the article (par 5.1)**

 The article reports on the order of forty distinct percentages, most to two significant figures, without a single case count. Par 3.4 and par 5.1 explain that the complete denominator record is unavailable, and I accept that the run-level record is lost. But the presentation still asserts a precision the archive cannot support: "100 percent accuracy" for reverse image search, "16 percent" for Know News, "42 percent" for The Factual in the multi-source analysis, where the denominator could be five cases or five hundred. The deposit constrains this in places: the only reduced labelled text subsets it retains are 32 and 42 items, and the social-media examples are three files of five rows each. If the fact-checking figures rest on samples of that order, that is material to a reader assessing the CT-A/CT-B divergence, and approximate values are presumably recoverable from the partners even without the logs. Report approximate n where it can be established, even as a range; where it genuinely cannot, report coarse bands instead of two-significant-figure percentages. This concedes nothing the article can legitimately claim, and it removes an implied precision that the rest of the revision has been careful to disclaim.

**MiC3: Residual sentences that contradict the revised framing**

 These are copy-level, but they matter, because a reader who meets them is entitled to infer that the qualifications elsewhere are pro forma.

 Par 3.4: "Accuracy was examined by comparing the tool's outputs with the expected results embedded in the synthetic datasets." This contradicts par 3.2, which states that "Not all retained material was generated in-house or is synthetic", and it occurs in the description of the accuracy measurement itself.

 Par 6, final sentence: "By offering a reproducible, ethically grounded, and operationally informed framework". This contradicts the first paragraph of the same section, "It therefore advances methodological transparency without claiming a fully reproducible benchmark", and the Abstract. It is also the sentence most likely to be quoted.

 Par 5.2, first sentence: "GPT-4o and The Factual were retained as the most accurate and scalable fact-checking instruments". The qualification arrives three paragraphs later, with the tool inventory in between, so a reader can take the recommendation and miss the condition. Add a forward pointer in the sentence itself.

 Par 5.1, Disinformation Campaign Analysis: "the findings confirm that while content-verification tools are mature and reliable". Par 4.1 now closes by stating that this maturity claim "should be read as conditional on the tested content resembling previously documented cases", but the conditioning is only forward referenced from par 4.1 and never applied at the place where the claim is made.

 Par 3, opening: "designed to provide a structured and reproducible process". The following sentences qualify it, but the contested word is doing unqualified work in the topic sentence.

 Data availability, third paragraph: "The available data package has been deposited in Zenodo (link above and at [32])". Reference 32 is Germani and Spitale on source framing; the deposit is reference 35.

 Par 6 places the incremental research agenda, that is machine-learning tools, pipeline orchestration and interoperability, immediately before the paragraph identifying the regulatory constraint as the more fundamental problem. Consider reordering so that the structural point is not read as an afterthought to the incremental one.

**MiC4: Author contributions and acknowledgements**

 The Amendments note states that author contributions were added. I could not locate an author-contributions statement in version 2, and the Acknowledgements still read "The author would like to thank" and "the author wishes to thank" for a seven-author article. Worth confirming that the section survived production.

**Recommendation: Approved with Reservations**

 The methodological contribution is real, and this revision brings the article's claims into line with the evidence that supports them. The two structural problems that made version 1 unacceptable have been addressed at the level at which they were raised, not deflected into limitations paragraphs. The "automation mentality" observation, which I described in my first report as the most accurate diagnosis in the article, now functions as part of its normative spine through the protocol in par 5.2 and the closing design principle in par 6, which is what I hoped would happen.

 MaC9 and MaC10 should be treated as required corrections rather than as suggestions. MaC9 because the deposit on which the credibility of this revision rests is not the one the article links to, and no reader can currently verify the corpus claims by following the reference given. MaC10 because publishing personal data under an open licence while the lawful basis for redistribution is marked as pending in the authors' own documentation is inconsistent with the standard the article itself defends in par 3.5 and par 5.3, and because the unconfirmed provenance of the text corpora blocks assessment of the contamination caveat that resolves MaC1(a). Both are fixable without touching the analysis. MaC11 requires one clarifying sentence and possibly a renaming of the instrument. The minor comments are improvements rather than conditions.

Is the rationale for developing the new method (or application) clearly explained?

Partly

Is the description of the method technically sound?

Partly

Are the conclusions about the method and its performance adequately supported by the findings presented in the article?

Partly

If any results are presented, are all the source data underlying the results available to ensure full reproducibility?

No

Are sufficient details provided to allow replication of the method development and its use by others?

No

Reviewer Expertise:

ethics, AI, disinformation, infodemic management

I confirm that I have read this submission and believe that I have an appropriate level of expertise to confirm that it is of an acceptable scientific standard, however I have significant reservations, as outlined above.

# Reviewer report: Reviewer response for version 1

**DOI:** 10.21956/openreseurope.23477.r74676

**Published:** 2026-07-06

**Authors:** Veysel Bozkurt

**Affiliations:** 1 Istanbul University, Istanbul, Turkey

Paper title: “Detecting FIMI: A methodological framework for testing OSINT tools in TTPs detection”

 Reviewer recommendation: Approved with Reservations

 The paper, developed under the Horizon Europe RESONANT project, presents a methodological framework for testing open-source intelligence (OSINT) tools aimed at detecting the tactics, techniques, and procedures (TTPs) used in FIMI (Foreign Information Manipulation and Interference) operations. The topic sits in an area that is highly current and carries real policy relevance. The lack of standardization in evaluating OSINT tools is a problem genuinely raised in the literature, and the authors' effort to approach it through a structured testing protocol deserves credit. Involving law enforcement partners from Greece and Ukraine in the testing process gives the study an operational grounding.

 The research problem is clearly defined. The observation that OSINT tool evaluation remains fragmented and unstandardized is accurate and matches practitioner experience in the field. The three-layered detection logic built on the DISARM, AMITT, and MITRE ATT&CK taxonomies (content-level verification, platform-level monitoring, infrastructure-level attribution) offers an analytically functional framework. Using synthetic data instead of live operational data is the right choice on ethical and legal grounds. The references to GDPR and DPIA procedures are handled with care. Having law enforcement experts assess usability and interpretability alongside technical accuracy, rather than accuracy alone, is a genuine contribution that sets this study apart from purely academic reviews. The honest reporting of the structural fragility caused by tool discontinuation, API restrictions, and platform policy changes is a valuable warning for researchers in this field.

 At the same time, the paper does not yet meet the core obligation expected of a method paper, namely describing the method at a level that others can apply and verify. The size, language, and construction procedure of the synthetic datasets are not reported. The performance indicators are defined conceptually but not operationalized at the level of measurement. Percentage values are presented without denominator information. The large discrepancies between the results of the two testing teams are left unexplained. Until these gaps are addressed, the article's claim to reproducibility remains unsupported.

 There is an internal inconsistency in the test design. Sections 3.2 and 3.3 state that each tool was exposed to identical inputs within its category, and that this is what makes the comparison meaningful. The opening paragraph of Section 5, however, states that the CT-A and CT-B teams tested the tools on different datasets and under their own operational conditions. These two statements contradict each other. The authors need to clarify the test design. Which tool was tested with which dataset, on how many observations, and by which team. Without this information, none of the findings in Section 5 can be properly assessed.

 The synthetic datasets are not described. The paper places synthetic datasets at the center of its methodology but provides almost no technical information about them. How many claims, images, accounts, and email addresses were generated. How were ground truth labels assigned. What language were the datasets in. How was difficulty calibrated. None of these questions is answered in the text. The language question is especially important, since the authors' own findings show weak multilingual processing capacity. Whether the test data was in English, Greek, or Ukrainian directly affects how the results should be read. The 100 percent accuracy achieved in reverse image search also points to a ceiling effect, suggesting the dataset may lack discriminative power and be too easy. The inability to share the datasets for security reasons is understandable, but nothing prevents reporting descriptive statistics and the construction protocol.

 The performance indicators are not operationalized. Accuracy, usability, interoperability, coverage, and sustainability are referred to as KPIs, yet none of them is defined at the level of measurement. Percentage values are given without denominators; a reader cannot tell whether a 90 percent accuracy figure comes from 10 observations or 500. There is no confidence interval, margin of error, or statistical test of any kind. No structured instrument (such as the System Usability Scale) was used for the usability assessment; the observations rest on free-form notes. Inter-rater reliability between the two teams was not calculated. A method paper exists precisely to document this kind of measurement protocol. The authors could prepare a table that specifies, for each KPI, an operational definition, a unit of measurement, and a data collection instrument.

 The contradictory findings across teams are left unexplained. In the CT-A evaluation, GPT-4o correctly classified 58 percent of false claims; in the CT-B evaluation, this figure was 95 percent. The 37-point gap between the two is left unexplained. Because the comparability of the datasets and the implementation protocols across teams is not documented, this 37-point discrepancy cannot be interpreted.

 The risk of training data contamination in the GPT-4o evaluation is overlooked. Declaring GPT-4o the most successful verification tool raises a serious validity concern. The synthetic claims were derived from known disinformation cases. There is a strong likelihood that these cases, along with the debunking content that refutes them, appear in GPT-4o's training data. What is being measured may therefore not be the tool's verification capacity but its capacity to recall training data. No conclusion about GPT-4o's superiority can be drawn without addressing this contamination risk. In addition, the test date, model version, prompt text used, and parameters such as temperature are not reported. Since large language models change behavior across version updates, this information is essential for reproducibility. It would also help to address the risk of hallucination and the limits of the model's ability to cite sources.

 The tool selection protocol is not transparent, and the claim of being “systematic” is not supported. The abstract uses the phrase “systematic multi-source collection,” and the introduction claims to offer “the first systematic state-of-the-art review.” Yet the text contains none of the elements expected of a systematic review, such as search strings, databases searched, inclusion and exclusion criteria, or a flow diagram. How the tools were found and how they were narrowed down cannot be traced. The selection protocol should either be reported transparently, or the label “systematic” should be removed from the text.

 There is tension between the reproducibility claim and the data access policy. The article presents reproducibility as one of its main contributions. At the same time, the datasets, scenarios, and testing materials are kept closed for security reasons; the Zenodo record contains only non-sensitive supporting documents. The rationale for restricted access may be legitimate, but in that case the scope of the reproducibility claim should be narrowed and reframed around a more defensible notion, such as the transferability of the methodological template. As it stands, there is a tension between the claim and the data access policy that readers will notice. Adding at least the dataset schemas, size information, and measurement protocol to the Zenodo record would considerably ease this tension.

 Conclusion and Recommendation

 The article addresses a significant practical problem and occupies a distinctive position through its operational partnerships. However, it has not yet reached the core purpose of a method paper, which is to offer a transferable and verifiable protocol. The manuscript cannot be approved in its current form until the internal inconsistency in the test design is resolved, the datasets and measurement protocol are documented, and the contamination risk in the GPT-4o finding is addressed. That said, all of these issues are correctable, and the core idea is worth preserving.

 My recommendation is Approved with Reservations. Once the points raised above are reasonably addressed, or the authors provide reasonable justification that satisfies the editor, I believe the article will reach an approvable standard. A further round of review should not be necessary. If the editor finds the revision satisfactory, the article can be accepted for indexing. I wish the authors success in the revision process.

Is the rationale for developing the new method (or application) clearly explained?

Partly

Is the description of the method technically sound?

Partly

Are the conclusions about the method and its performance adequately supported by the findings presented in the article?

Partly

If any results are presented, are all the source data underlying the results available to ensure full reproducibility?

No

Are sufficient details provided to allow replication of the method development and its use by others?

No

Reviewer Expertise:

Sociology of Work/Social Impacts of AI

I confirm that I have read this submission and believe that I have an appropriate level of expertise to confirm that it is of an acceptable scientific standard, however I have significant reservations, as outlined above.

# Reviewer report: Reviewer response for version 1

**DOI:** 10.21956/openreseurope.23477.r75053

**Published:** 2026-06-17

**Authors:** Marius Dragomir

**Affiliations:** 1 University of Santiago de Compostela, Santiago de Compostela, Spain

This article presents a methodological framework, developed within the Horizon Europe RESONANT project, for testing open-source intelligence (OSINT) tools used to detect tactics, techniques, and procedures (TTPs) associated with Foreign Information Manipulation and Interference (FIMI). The article reports per-tool performance observations, proposes an "optimized tool suite," and reflects on cross-cutting limitations including API volatility, weak interoperability, limited multilingual coverage, and the legal and ethical constraints of OSINT work. The topic is timely and important, the conceptual framing is appropriate, and the involvement of operational partners gives the study practical relevance beyond a purely academic tool review.

 The rationale for the work is clearly and convincingly explained, which is why I have answered "Yes" to the first question. The authors establish that OSINT tool evaluation in the FIMI domain is fragmented and lacks standardization, and they motivate the shift from narrative-level analysis to TTP-oriented detection well. The grounding in DISARM, AMITT, and MITRE ATT&CK is appropriate, and the relevance to researchers, law enforcement, and EU policy audiences is articulated effectively. No revision is required on this point, in my view.

 I have answered "Partly" to whether the method is technically sound because the overall logic is coherent, but the specification has gaps that I believe should be closed. The "Waterfall model" is invoked as the methodological inspiration but functions largely as a label; the authors should either demonstrate concretely how the stepwise structure shaped the testing design and traceability, or reduce this framing, since as written it adds citations without analytical payoff. More importantly, the operationalization of the TTP frameworks is underspecified: the authors should explain precisely how tools and test cases were mapped to specific DISARM, AMITT, and ATT&CK tactics and techniques. The manuscript also conflates three distinct claims that should be kept separate (whether a tool detects a TTP, produces evidence relevant to a TTP, or merely supports a broader investigative task), which represent very different evidentiary standards and should not be reported interchangeably. The evaluation indicators are also described conceptually rather than operationally, and there is a notable inconsistency between the manuscript and the project's own deposited materials: the article describes its indicators as accuracy, usability, interoperability, coverage of adversarial behaviors, and sustainability, whereas the deposited metrics file defines seven evaluation metrics on 1–5 ordinal scales (accuracy, coverage, speed/performance, ease of use, community feedback/satisfaction, customization/configurability, and support/documentation) that do not cleanly map onto the five indicators named in the text, and do not clearly include interoperability or sustainability. The deposited materials further indicate that ease-of-use was captured through a System Usability Scale instrument with only one response per tool per team, so usability findings have no inter-rater basis unless an internal consensus procedure is described. The authors should reconcile the indicator sets, report results in the units actually used, state how each indicator was scored and by whom, and acknowledge the single-rater limitation.

 I have answered "No" to whether sufficient details are provided to allow replication, and this is the most serious deficiency in my view. The paper claims a "systematic multi-source collection" of tools but provides no search strategy, source list, search dates, inclusion or exclusion criteria, number of tools screened versus retained, or rationale for excluding particular tools; as a result, the descriptor "systematic" is insufficiently documented (the authors may have followed a systematic process internally, but the article does not record it). They should add a transparent selection protocol (PRISMA-style or an equivalent structured account) and a final tool inventory with version numbers and testing dates. The synthetic datasets, which carry much of the study's validity, are described only in general terms: the authors should specify how each case was generated, how cases were mapped to specific TTPs, how realism and difficulty were calibrated, whether any real-world FIMI incidents served as anchors, whether domain experts validated the cases and annotations, and how many cases were used per category. The testing procedure also needs detail, and here the manuscript appears internally inconsistent: the methodology states that tools within a category were applied to the same dataset, implying category-level comparability, whereas the results section states that CT-A and CT-B each tested tools on distinct datasets under their respective operational conditions. The authors should clarify whether the two cyberteams tested identical or distinct datasets, since the reported percentages cannot be directly compared if the inputs differed. The procedure should also state how evaluator observations were captured and whether a shared rubric, consensus procedure, or measure of inter-rater agreement was used. In the publicly available Zenodo materials I examined, the open files document the intended evaluation framework and tool inventory but not the completed testing records; the logging template is empty. The deposit therefore documents the intended method without evidencing its execution and cannot substitute for the procedural detail requested here. These points should be addressed for the article to be scientifically sound as a method article.

 I have answered "Partly" to the availability of source data, having examined the open Zenodo deposit (DOI 10.5281/zenodo.19045753. In the publicly available materials I examined, the deposit contains two files: a metrics document defining the evaluation indicators and their 1–5 scoring scales, together with the list of tools and the metric categories intended for each tool; and a documentation template in spreadsheet form. This makes the evaluation instrument and the tool inventory transparent, which is welcome and should be credited. However, the deposit does not contain the data underlying the reported results. The documentation spreadsheet appears to be an empty template, its logging sheet (columns for scenario number, tool, dataset, testing date and time, observed output, and issues faced) and its usability questionnaire are unfilled, and the synthetic datasets that generated the reported figures are, by the authors' own statement, restricted for security reasons. Consequently, none of the quantitative results presented in the article, namely the per-tool accuracy percentages, can be traced to deposited source data. A further misalignment compounds this: the deposited metrics file defines seven evaluation metrics on 1–5 ordinal scales, but these do not fully align with the five indicators described in the manuscript, and the article's results are reported mainly as accuracy percentages rather than in the metric units defined in the deposited framework. To address the reproducibility claim, the authors should deposit the completed logs and scored results (suitably anonymized), reconcile the reported metrics with the framework actually used, and either release a sanitized subset of the synthetic datasets with their per-case TTP mappings or temper the reproducibility language accordingly.

 I have answered "Partly" to whether the conclusions are adequately supported. The qualitative conclusions (that no single tool or category is sufficient, that content-verification and infrastructure-verification tools are relatively mature, and that bot detection and social media analytics are structurally fragile) are reasonable and broadly consistent with the reported observations. The difficulty lies with the quantitative claims and the tool recommendations. The results report many precise percentages, but the denominator is rarely given, ground-truth establishment is unspecified, and false-positive and false-negative definitions are absent; the authors should state, per category, the number of test cases, how ground truth was set, and how errors were classified. Several figures cannot currently be interpreted—for instance, an email-verification tool identifying "21–22 percent of valid addresses" is described as the most consistent performer, which is uninterpretable without the denominator and a definition of success. This reporting issue needs to be addressed, and it is compounded by the fact that none of these figures appears in the deposited documentation, and none is expressed in the 1–5 metric framework the project defined. A specific case requiring attention is the evaluation of generative AI systems: the article reports GPT-4o as a high-performing fact-checking instrument (around 90–95 percent accuracy), but where such systems are evaluated, the authors should report the exact model version, access date, prompt protocol, settings, retrieval or browsing conditions, number of runs, and the procedure for handling output variability; without these, the results are not independently reproducible, particularly as model behavior and product configuration can change over time. Finally, the proposed "optimized tool suite" includes commercial, proprietary, and platform-dependent tools whose functionality changes rapidly, and the selection criteria are not always transparent; the authors already acknowledge that results are time-bound, and they should make this caveat explicit in the recommendations themselves rather than presenting the suite as generally valid beyond the testing window.

 In summary, the article makes a genuine and policy-relevant contribution and rests on a strong rationale, but it is not yet sufficiently specified to support its central claim as a reproducible methodological framework. The essential revisions concern transparency and reporting rather than the underlying work and are probably achievable quite easily.

 The points that should be addressed in my view to make the article scientifically sound are those underlying the "No" answer on replication and the performance-reporting gaps under the conclusions question; on source data, the open deposit provides the instrument and tool list but none of the completed results or datasets that underlie the reported figures, and the reported percentages are neither documented in the deposit nor expressed in the metric framework the project defined.

Is the rationale for developing the new method (or application) clearly explained?

Yes

Is the description of the method technically sound?

Partly

Are the conclusions about the method and its performance adequately supported by the findings presented in the article?

Partly

If any results are presented, are all the source data underlying the results available to ensure full reproducibility?

Partly

Are sufficient details provided to allow replication of the method development and its use by others?

No

Reviewer Expertise:

Disinformation, Media literacy, Media Studies, Broadcast regulation, PSM, Audience studies, Media sustainability,

I confirm that I have read this submission and believe that I have an appropriate level of expertise to confirm that it is of an acceptable scientific standard, however I have significant reservations, as outlined above.

# Reviewer report: Reviewer response for version 1

**DOI:** 10.21956/openreseurope.23477.r74680

**Published:** 2026-06-15

**Authors:** Giovanni Spitale

**Affiliations:** 1 IBME, Universitat Zurich, Zurich, Switzerland

The paper presents a structured methodology for evaluating OSINT tools against FIMI-related TTPs, developed within the Horizon Europe RESONANT project. The study tests eight tool categories using synthetic datasets and law enforcement practitioners as evaluators, mapping outputs to DISARM, AMITT, and MITRE ATT&CK frameworks. The methodological contribution is genuine: reproducible, ethically compliant testing of a fragmented tool ecosystem is a real gap, and the paper fills it competently. However, several structural problems undermine the reliability of the central empirical claim, the scope of the ethical analysis, and the normative adequacy of the proposed tool suite. These are detailed below.

 Major Comments

 MaC1: GPT-4o performance figures are not interpretable as stated (par5.1, fact-checking paragraph)

 "GPT-4o consistently outperformed all other tools, correctly identifying around 90 percent of true and 58 percent of false claims in the CT-A tests, and achieving 94 percent accuracy on true and 95 percent on false claims in the CT-B evaluation"

 This is the most consequential empirical finding in the paper, and it is reported without the information needed to assess it. Three problems:

 (a) The synthetic dataset is circular with respect to LLM evaluation. The authors describe synthetic content as "fabricated narratives derived from known disinformation cases" (par 3.2). GPT-4o was trained on exactly this type of known, publicly documented disinformation. The model's high accuracy on these inputs does not necessarily demonstrate fact-checking capability; rather, it might demonstrate recall of training data. The test would need to be conducted on novel, out-of-distribution content (information voids, emerging narratives, non-anglophone campaigns) to support the claim that GPT-4o "represents the most reliable fact-checking instrument." This distinction is acknowledged obliquely in par 6: "reliance on synthetic datasets […] cannot fully capture the dynamic unpredictability of evolving information manipulation tactics", but the implication for the par 5.1 performance figures is not drawn. The conclusion in par 5.2 that GPT-4o should be "retained as the most accurate and scalable fact-checking instrument" follows only if the synthetic dataset is a valid proxy for operational conditions. It is not, I believe, and the paper should say so explicitly here rather than in a limitations paragraph that is structurally separated from the results. I recommend either to re-run the study on meaningful data or at least add a paragraph in par 5.1 explicitly addressing the circularity risk for LLM-based fact-checkers tested on synthetic data derived from known cases.

 (b) No inter-rater reliability is reported for ground truth labelling. The synthetic dataset was produced internally by the RESONANT project (par 3.2). No information is given about how ground truth labels were assigned or validated. The accuracy figures in par 5.1 are ratios against these labels. Without inter-rater reliability measures, the accuracy figures are uninterpretable. I recommend reporting ground truth labelling procedure and inter-rater reliability.

 (c) The large performance gap between CT-A and CT-B for the same tool (GPT-4o: 58% false-claim detection in CT-A vs. 95% in CT-B) is unexplained. A 37-percentage-point difference on false claims for an identical tool suggests that either the two synthetic datasets are not equivalent, or the evaluation criteria differed. The paper notes that teams "tested tools on distinct datasets under their respective operational conditions" (par 5 preamble) but does not analyze or explain what drives this divergence or what it implies for the methodological comparability the study claims. I recommend explaining the CT-A/CT-B performance divergence for GPT-4o or acknowledge it as a structural limitation that bounds the reliability of the comparative rankings.

 (d) The CT-A/CT-B comparison is underpowered as a validation strategy (par 5 preamble, par 5.1)

 Using two teams on distinct datasets as a validation strategy conflates two sources of variance: team-level variation (skills, interpretation, operational procedures) and dataset-level variation (different synthetic content). The 100% vs. 43% accuracy difference for Search by Image in the Disinformation Campaign Analysis (par 5.1) and the 0% vs. 100% difference for The Factual in the Cross-Platform analysis are not interpretable at all under this design. The study cannot distinguish whether these differences reflect tool instability, dataset differences, or evaluator differences. This does not invalidate the methodology, but it means the performance figures in par 5.1 cannot be treated as reliable performance estimates, only as existence proofs that tools can produce these outputs under at least one configuration.

 MaC2: Known systematic bias of GPT-4o on information evaluation tasks is not acknowledged (par 5.1, par 5.2)

 "GPT-4o and The Factual currently represent the most reliable fact-checking instruments" (par 5.1)

 "GPT-4o and The Factual were retained as the most accurate and scalable fact-checking instruments" (par 5.2)

 The recommendation to integrate GPT-4o as the primary fact-checking instrument in a FIMI detection suite for law enforcement is made without any reference to the documented systematic biases of large language models in exactly this evaluative task.

 References demonstrated that the declared source of a text, independent of its content, systematically shifts LLM credibility scores. This bias is structural and reflects regularities in training data and cannot be corrected by prompting alone. Similar ideological and political biases have been widely documented in literature; https://link.springer.com/article/10.1007/s42001-025-00376-w; https://news.mit.edu/2024/study-some-language-reward-models-exhibit-political-bias-1210 ; https://cs191w.stanford.edu/projects/Huang,%20Flora_CS191.pdf ; https://aclanthology.org/2024.emnlp-main.508/   ; https://dl.acm.org/doi/abs/10.1145/3461702.3462624 ; https://www.degruyterbrill.com/de/document/doi/10.1515/jtc-2023-0019/html ; https://arxiv.org/abs/2405.16455  ). argues that the opacity of these biases precludes the calibrated trust that responsible use of any epistemic authority requires.

 In the FIMI detection context, this has direct operational implications. Deploying a tool with structural known and documented source-attribution bias for attribution-related judgments is a threat to the validity of the detection paradigm the paper proposes. The authors mention in par 5.3 that "non-transparent scoring systems" reduce tool value, but do not apply this observation to GPT-4o, which is the most opaque tool in the entire suite.

 IMHO Par 5.2 should include a paragraph on the source-framing bias documented in LLMs and its implications for GPT-4o's suitability as a neutral fact-checking instrument in geopolitically sensitive FIMI contexts. This does not require removing GPT-4o from the suite, but it requires qualifying the recommendation and specifying operational safeguards (e.g., instructions to evaluate content without source information presented to the model, cross-validation with source-agnostic tools, …).

 MaC3: The ethics section conflates legal compliance with epistemic ethics (par 3.5, par 5.3)

 "All testing activities were conducted in line with the GDPR and under the project's DPIA. By using synthetic datasets and open-source tools, the methodology avoided high-risk data processing." (par 3.5)

 "Analysts must ensure that their actions are necessary, proportionate and respectful of fundamental rights. This includes avoiding techniques that could be seen as intrusive, manipulative, or misleading." (par 5.3)

 The ethical framework deployed in this paper is entirely juridical: GDPR compliance, proportionality, data minimization. This is appropriate for research ethics but insufficient for a paper that proposes deploying LLM-based verdict tools in law enforcement FIMI investigations.

 The paper does not engage with the distinct ethical problem of algorithmic judgment replacing human judgment in epistemic contexts. When GPT-4o flags a claim as likely false and this output feeds into a law enforcement investigation, the model functions as an epistemic authority. The opacity of that authority — its inability to provide reasons in principle open to scrutiny and contestation — is an ethical problem that GDPR compliance does not address. The same paragraph that mentions "non-transparent scoring systems" as a usability issue (par 5.3) should recognize that non-transparency in verdict-producing tools is an epistemic ethics issue, not merely a UX issue.

 Relatedly, the "automation mentality" paragraph in par 5.1 ("OSINT tools are simple, requiring only pressing a button and wait for the results [...] the tools can assist in the intelligence gathering process, but they cannot replace human creativity, analytical thinking, and reasoning") is the most intellectually honest passage in the paper, but it sits in isolation. It names the correct structural risk and then drops it. This risk should be integrated into the normative framing of the proposed tool suite in par 5.2 and the conclusions in par 6, not treated as a peripheral cautionary note.

 I suggest that par 5.3 should distinguish between legal-compliance ethics (GDPR, DPIA) and epistemic ethics (the question of what kinds of algorithmic output are appropriate bases for investigative judgment). The "automation mentality" observation should be carried forward into the tool suite recommendations in par 5.2: which tools in the proposed suite produce verdicts, which produce structured evidence that analysts interpret, and what procedures govern when each type of output can be used operationally?

 MaC4: The "content-level" detection paradigm's foundational limitation is not theorized (par 2.1, par 3.1, par 5.1)

 "By focusing on the 'how' rather than the 'what' of disinformation, TTP frameworks enable analysts to identify recurring patterns, compare incidents, and design countermeasures." (par 2.1)

 "Aligning tool functions with these frameworks ensured that the review addressed the full spectrum of TTPs relevant to FIMI detection" (par 3.1)

 The paper correctly frames TTPs as structural rather than content-dependent at the taxonomic level (DISARM, AMITT). But the actual tools evaluated (fact-checkers, credibility databases, bot detectors) are almost entirely content-dependent in operation: they match specific claims against existing records, assess known domains, or detect known behavioral signatures. The paper notes that "novel narratives emerge" as a limitation of fact-checking tools (par 4.1) but does not recognize this as a fundamental paradigmatic limitation of the entire content-level detection layer.

 Research on viral misinformation detection has demonstrated that content-based systems fail systematically on information voids, precisely the events (crises, novel geopolitical developments) where FIMI operations are most dangerous and most active. The failure is not contingent on dataset quality or tool maintenance; it is structural. A methodology that presents GPT-4o and The Factual as the best fact-checking instruments must acknowledge that both tools depend on having encountered the relevant claims (in training data or indexed databases) before they can evaluate them. For genuinely novel FIMI content, the entire content-verification layer of the proposed suite degrades to near-zero.

 The paper's conclusion that "content-verification tools are mature and reliable" (par 5.1, Disinformation Campaign Analysis paragraph) should be qualified to specify the conditions under which this holds.

 Par 2.1 or par 4.1 should include a paragraph contrasting content-dependent and content-agnostic detection paradigms in case you feel you need theoretical backing on content agnostic paradigms. The structural limitation of content-based approaches for novel-content scenarios should be explicitly stated in par 5.1 and carried forward into the future research directions in par 6.

 MaC5: The proposed tool suite has no normative architecture (par 5.2)

 "No single tool or category was sufficient to address the full spectrum of adversarial tactics, techniques, and procedures. However, by combining high-performing instruments across content, platform, and infrastructure levels, it is possible to build a detection ecosystem that compensates for individual weaknesses." (par 5.2)

 The optimised suite is presented as a technical aggregation of best-performing tools across layers. What is missing is any normative specification of how the suite should be operated, in particular, what procedural constraints govern the use of verdict-producing outputs (GPT-4o's fact-check verdicts, credibility scores) as inputs to investigative or attributional processes.

 The distinction between tools that produce structured evidence for analyst interpretation versus tools that produce verdicts intended for direct use is consequential in a law enforcement context. The paper treats this as a usability question ("unclear user interfaces, non-transparent scoring systems") but it is operationally and legally a question about the weight that different output types can carry. The absence of a proposed decision protocol (for example, specifying that GPT-4o fact-check outputs should require confirmation from a second independent source before informing any investigative action) means the "optimised suite" is a list of tools, not a framework for their use.

 I believe par 5.2 should include at least a brief decision protocol specifying, at minimum: (a) which tool outputs are to be treated as evidence for further investigation versus conclusions; (b) which outputs require cross-validation before operational use; (c) conditions under which human review must override automated outputs.

 MaC6: The "FIMI" label conflates analytically distinct phenomena (par 1, throughout)

 The paper uses FIMI as a unified analytical category throughout. Within the detection context, however, the distinction between misinformation (false content produced without intent to deceive) and disinformation (false content produced with strategic intent) has direct operational implications: they require different attributional procedures and map differently onto legal responsibility frameworks. The paper acknowledges this implicitly (the TTP-based approach focuses on how operations are conducted, which is closer to disinformation (intentional, strategic)). But fact-checking tools (par 4.1, par 5.1) detect what is false, which is intent-agnostic. The conflation leads to inconsistencies: in par 3.2, the synthetic datasets include "fabricated narratives" (disinformation-type) alongside what appear to be simple false claims tested against fact-checking repositories (misinformation-type). Clarifying which part of the tool suite addresses which type of epistemic harm would sharpen both the methodology and the conclusions.

 MaC7: The "ethics by design" claim is asserted but not operationalized (par 3.5)

 "The approach was also guided by ethics-by-design principles, ensuring that AI- and NLP-based detection methods were assessed with attention to transparency, explainability and potential bias."

 This sentence appears in par 3.5 but has no corresponding operationalization anywhere in the results or discussion. No bias assessment is reported for any AI-based tool. No transparency metric is defined or measured. No explainability criterion appears in the KPIs described in par 3.4, which cover accuracy, usability, interoperability, coverage, and sustainability. The "ethics by design" reference reads as rhetorical assurance rather than a methodological commitment. Either the bias/explainability assessment should be reported as a result, or the claim should be removed from par 3.5.

 MaC8: Future research directions miss the most pressing structural gap (par 6)

 "Future studies could expand the testing protocol to integrate machine-learning-driven tools, automated pipeline orchestration and cross-platform detection capabilities." (par 6)

 The future research agenda proposed in par 6 extends the existing paradigm (more automation, more tools, more interoperability). It does not address the structural problem the paper's own results document: the two most critical detection categories (i.e. bot detection and social media analytics) are essentially non-functional due to platform API restrictions, and no technical solution to this is conceivable within the current third-party tool model. The real structural question, I.e. whether FIMI detection at scale requires public regulatory access to platform data, not better third-party tools, is not even named. The conclusion that these categories require "renewed development of sustainable, API-driven, and institutionally supported solutions" (par 5.1) is correct but stops short of the regulatory implication.

 Recommendation: (very) Major revision

 The paper addresses a genuine methodological gap: the OSINT tool ecosystem for FIMI detection is fragmented, and structured reproducible evaluation frameworks are scarce. The layered approach (content, platform, infrastructure), the practitioner-validated testing environment, and the explicit alignment with established TTP taxonomies are real contributions.

 However, the paper in its current form cannot be accepted for two compounding reasons. First, the central empirical claim (that GPT-4o represents the most reliable fact-checking instrument and should anchor the proposed detection suite) rests on performance figures that are not interpretable as stated (MaC1: synthetic dataset circularity, absent inter-rater reliability, unexplained 37-point CT-A/CT-B divergence) and ignores a body of published evidence on systematic LLM bias in exactly the evaluative context the study proposes to deploy it (MaC2). Second, the normative framework is structurally incomplete: the ethics sections address GDPR compliance exclusively, while the paper proposes deploying verdict-producing algorithmic tools in law enforcement investigations without any decision protocol governing their use (MaC3, MaC5). The "automation mentality" paragraph in par 5.1 correctly names the core risk and then abandons it; this is the most accurate diagnosis in the paper and it should be its normative spine, not a footnote.

 The remaining comments (MaC4 on content-dependence, MaC6 on the mis/disinformation conflation, MaC7 on the unoperationalized ethics-by-design claim, MaC8 on the regulatory gap in future directions) are substantive but addressable without new data collection. MaC1 and MaC2, however, require either re-analysis or a fundamental re-scoping of what the performance results are claimed to demonstrate.

 The authors should be encouraged to revise. The methodological skeleton is sound, the empirical work is real, and the operational context (law enforcement, Horizon Europe) gives the paper genuine policy relevance. But it needs to be honest about what its data can and cannot support, and it needs to take the ethics of algorithmic judgment in investigative contexts as seriously as it takes GDPR compliance.

Is the rationale for developing the new method (or application) clearly explained?

Partly

Is the description of the method technically sound?

Partly

Are the conclusions about the method and its performance adequately supported by the findings presented in the article?

Partly

If any results are presented, are all the source data underlying the results available to ensure full reproducibility?

No

Are sufficient details provided to allow replication of the method development and its use by others?

No

Reviewer Expertise:

ethics, AI, disinformation, infodemic management

I confirm that I have read this submission and believe that I have an appropriate level of expertise to state that I do not consider it to be of an acceptable scientific standard, for reasons outlined above.

## References

[1] : Source framing triggers systematic bias in large language models. *Science Advances* .2025;11(45) : 10.1126/sciadv.adz2924 10.1126/sciadv.adz2924 PMC1314276441202130

[2] : The political preferences of LLMs. *PLOS ONE* .2024;19(7) : 10.1371/journal.pone.0306621 10.1371/journal.pone.0306621 PMC1129062739083484

[3] : The political preferences of LLMs. *PLOS ONE* .2024;19(7) : 10.1371/journal.pone.0306621 10.1371/journal.pone.0306621 PMC1129062739083484

[4] : Assessing political bias in large language models. *Journal of Computational Social Science* .2025;8(2) : 10.1007/s42001-025-00376-w 10.1007/s42001-025-00376-w

[5] : Cultural Bias in Large Language Models: A Comprehensive Analysis and Mitigation Strategies. *Journal of Transcultural Communication* .2025;3(2) : 10.1515/jtc-2023-0019224-244 10.1515/jtc-2023-0019

[6] : Expertise, opacity, and trust in AI systems. *Synthese* .2026;207(3) : 10.1007/s11229-026-05484-2 10.1007/s11229-026-05484-2

[7] : The structural fingerprints of disinformation: a content-agnostic framework. *Ethics and Information Technology* .2026;28(2) : 10.1007/s10676-025-09886-7 10.1007/s10676-025-09886-7

# Reviewer report: Reviewer response for version 1

**DOI:** 10.21956/openreseurope.23477.r75059

**Published:** 2026-06-05

**Authors:** Kokil Jaidka

**Affiliations:** 1 National University of Singapore, Singapore, Lower Kent Ridge Rd, Singapore

This article presents a methodological framework for evaluating OSINT tools for the detection of tactics, techniques, and procedures associated with Foreign Information Manipulation and Interference. The framework is developed within the RESONANT project and combines tool mapping, synthetic datasets, controlled testing by practitioner teams, and evaluation across dimensions such as accuracy, usability, interoperability, coverage, and sustainability.

 My assessment focuses on the methodological, conceptual, and socio-technical dimensions of the study. The article has several strengths:

 - The rationale for the study is clear and timely. The paper identifies an important gap in the FIMI and OSINT ecosystem: although many tools exist for fact-checking, multimedia verification, bot detection, social media analysis, and infrastructure-level lookup, their evaluation remains fragmented and insufficiently standardised. The move from narrative-level disinformation analysis to TTP-oriented detection is also well motivated. The use of DISARM, AMITT, and MITRE ATT&CK as conceptual anchors is appropriate, and the article is relevant to researchers, law enforcement agencies, and EU policy audiences.

 - It usefully organises the OSINT tool landscape into content-level, platform-level, and infrastructure-level detection layers. It also gives appropriate attention to practical constraints that often shape real OSINT work, including API volatility, discontinued tools, subscription barriers, limited multilingual coverage, lack of interoperability, and the need for human judgment. The involvement of law enforcement partners from Greece and Ukraine gives the study operational relevance and helps move the discussion beyond a purely academic tool review.

 However, the manuscript requires substantial revision before it can support its central claims as a method article. My main concerns are:

 - The method is described at a relatively high level but is not specified in enough detail for replication or independent assessment. The paper states that tools were collected through a systematic multi-source process, but it does not provide a sufficiently transparent search strategy, inclusion and exclusion criteria, number of tools screened, final tool list with versions, testing dates, access conditions, or rationale for excluding particular tools. Without these details, the tool review cannot be assessed as systematic or reproducible.

 - The details on the construction and validation of the synthetic datasets are unclear. The paper states that the datasets included fabricated narratives, manipulated and AI-generated images, simulated bot activity, and spoofed infrastructure elements. This is a reasonable strategy for avoiding privacy, ethical, and operational risks. However, the manuscript does not provide enough detail about how these cases were generated, how they were mapped to specific TTPs, how realistic or difficult they were, whether experts validated them, or whether any real-world FIMI cases were used as anchors. Synthetic datasets can support controlled evaluation, but their validity depends heavily on their realism, diversity, and annotation quality.

 - The performance analysis needs much stronger reporting. The paper reports many percentages for individual tools, including GPT-4o, The Factual, Google Images, OCR tools, Geo-IP tools, phone verification tools, and email verification tools. However, the denominator is often unclear. The paper should state how many test cases were used for each category, how ground truth was established, how false positives and false negatives were defined, and whether the same inputs were used across both cyberteams. It should also explain how evaluator observations were recorded and whether any inter-rater agreement, consensus procedure, or structured rubric was used. At present, it is difficult to determine whether the results are robust evaluation findings or descriptive observations from practitioner testing.

 - The claim of reproducibility is not fully supported. The article states that the full synthetic scenarios, detailed dataset structures, and related testing materials are restricted. This may be justified for security reasons, but it creates a tension with the article’s repeated claims that the framework enables reproducible testing. A method article should provide enough procedural detail for another team to understand and apply the method with minimal ambiguity.

 - The distinction between method, results, and recommendations should be clarified. The article moves from tool descriptions to tool performance and then to an “optimised tool suite,” but the basis for recommending particular tools is not always sufficiently transparent. This is especially important because some of the recommended tools are commercial, proprietary, subscription-based, dependent on platform access, or subject to rapid changes in functionality. The authors should avoid presenting tool rankings as generally valid beyond the specific testing period unless the evaluation design supports that claim.

 The manuscript would also benefit from clearer operationalisation of the TTP frameworks. DISARM, AMITT, and MITRE ATT&CK are appropriate references, but the paper should explain more precisely how they were used in testing. Were tools mapped to specific tactics and techniques? Were synthetic test cases designed to correspond to particular TTPs? Did evaluators assess whether a tool could detect a TTP, produce evidence relevant to a TTP, or simply support a broader investigative task? These are distinct claims and should not be conflated.

 In conclusion, this is a useful and policy-relevant article, but it is not yet sufficiently specified as a methodological framework. The rationale is strong, and the topic is important. However, the current version does not provide enough methodological transparency, source-data availability, or performance-reporting detail to allow independent replication or full assessment of the findings. 

Is the rationale for developing the new method (or application) clearly explained?

Yes

Is the description of the method technically sound?

Partly

Are the conclusions about the method and its performance adequately supported by the findings presented in the article?

Partly

If any results are presented, are all the source data underlying the results available to ensure full reproducibility?

No

Are sufficient details provided to allow replication of the method development and its use by others?

No

Reviewer Expertise:

misinformation, computational linguistics, discourse analysis

I confirm that I have read this submission and believe that I have an appropriate level of expertise to confirm that it is of an acceptable scientific standard, however I have significant reservations, as outlined above.
